# Stimulated Electrocatalytic Hydrogen Evolution Activity of MOF‐Derived MoS_2_ Basal Domains via Charge Injection through Surface Functionalization and Heteroatom Doping

**DOI:** 10.1002/advs.201900140

**Published:** 2019-05-29

**Authors:** Gamze Yilmaz, Tong Yang, Yonghua Du, Xiaojiang Yu, Yuan Ping Feng, Lei Shen, Ghim Wei Ho

**Affiliations:** ^1^ Department of Electrical and Computer Engineering National University of Singapore 4 Engineering Drive 3 Singapore 117583 Singapore; ^2^ Department of Physics National University of Singapore Singapore 117551 Singapore; ^3^ Institute of Chemical and Engineering Sciences A*STAR (Agency for Science, Technology and Research) 1 Pesek Road Jurong Island Singapore 627833 Singapore; ^4^ Singapore Synchrotron Light Source National University of Singapore 5 Research Link Singapore 117603 Singapore; ^5^ Department of Mechanical Engineering National University of Singapore Singapore 117575 Singapore; ^6^ Institute of Materials Research and Engineering A*STAR (Agency for Science, Technology and Research) 3 Research Link Singapore 117602 Singapore

**Keywords:** basal plane, electrocatalysts, hydrogen evolution reaction (HER), metal–organic framework, MoS_2_

## Abstract

The design of MoS_2_‐based electrocatalysts with exceptional reactivity and robustness remains a challenge due to thermodynamic instability of active phases and catalytic passiveness of basal planes. This study details a viable in situ reconstruction of zinc–nitrogen coordinated cobalt–molybdenum disulfide from structure directing metal–organic framework (MOF) to constitute specific heteroatomic coordination and surface ligand functionalization. Comprehensive experimental spectroscopic studies and first‐principle calculations reveal that the rationally designed electron‐rich centers warrant efficient charge injection to the inert MoS_2_ basal planes and augment the electronic structure of the inactive sites. The zinc–nitrogen coordinated cobalt–molybdenum disulfide shows exceptional catalytic activity and stability toward the hydrogen evolution reaction with a low overpotential of 72.6 mV at −10 mA cm^−2^ and a small Tafel slope of 37.6 mV dec^−1^. The present study opens up a new opportunity to stimulate catalytic activity of the in‐plane MoS_2_ basal domains for enhanced electrochemistry and redox reactivity through a “molecular reassembly‐to‐heteroatomic coordination and surface ligand functionalization” based on highly adaptable MOF template.

Molybdenum disulfide (MoS_2_) has emerged as a promising hydrogen evolution electrocatalyst substitute for platinum (Pt) owing to its high catalytic activity, high structural stability, and cost‐effectiveness.^1^, ^2^, ^3^ As predicted by theoretical studies and verified by numerous subsequent experiments, hydrogen evolution reaction (HER) activity of MoS_2_ originates from the high catalytic activity of the Mo‐terminated edge sites, while the basal planes, which constitute the majority of the structure, are catalytically inactive.^2^, ^4^ Various approaches have been proposed to engineer MoS_2_ structure with the aim of increasing the density of active sites. One approach is morphology engineering, which focuses on maximizing the exposure of edge sites through nanostructuring.^5^, ^6^, ^7^, ^8^, ^9^ However, catalytic activity of these nanostructures is found to be restricted by the availability of the edge sites in the structure, whose density is generally less significant relative to that of the basal planes. There exists an approach to fully utilize the basal planes by phase transformation from the semiconducting 2H‐phase into metallic 1T‐phase.^10^, ^11^ Theoretical studies showed that such transformation radically alters the naturally inert basal planes into metallic active sites with an optimum hydrogen adsorption Gibbs free energy (Δ *G*_H_ = +0.18 eV).^11^, ^12^ Yet, 1T‐phase is thermodynamically metastable with a relaxation energy of ≈1.0 eV for conversion to the stable 2H‐phase, which hinders its further exploitation in HER application.^11^ Meanwhile, it has been demonstrated that unfavorable 2H‐MoS_2_ basal planes can also be exploited via electronic coupling with a conductive substrate that allows electron injection from the substrate to the catalytic sites.^13^, ^14^ Such electron transport promotes the rate‐determining Volmer reaction, therefore accelerates the kinetics of HER. However, it remains a challenge to minimize the contact resistance that leads to the formation of Schottky barrier for electron injection.

Rather than modifying the electronic properties of basal planes through interfacing with conductive substrates, direct intact electronic modulation could be an efficient strategy for activation and optimization of MoS_2_ electrocatalyst for hydrogen evolution.^15^, ^16^, ^17^, ^18^, ^19^ By breaking the periodicity in MoS_2_ crystal and reconfiguring it with alien atoms, it is possible to create localized electronic density on host atoms, hence altering the energy barriers for the reaction. This phenomenon is analogous to heteroatoms doping into graphene, where charge redistribution is realized on in‐plane carbon atoms adjoined to foreign atoms that greatly influences the catalytic activity.^20^, ^21^, ^22^, ^23^ Electronic modulation could attain an optimized matching between the electronic states of the active centers of the catalyst and interacting hydrogen. Molecular orbital theory studies have demonstrated that a meticulous charge regulation can tune the energy level of antibonding states, thereby can optimize the adsorption strength of hydrogen. Besides heteroatom doping, modification of the coordination environment has also a paramount importance in stimulating the activity of MoS_2_ in‐plane atoms. Recent studies have shown that electronic structure, surface chemistry, electrocatalytic reactivity, and stability of MoS_2_ can be modulated via conjugating the basal planes with ligands having different electron affinities.^24^, ^25^ However, decrease in HER performance was reported due to conjugation of the functional groups on edge and sulfur vacancy sites of complex MoS_2_ morphologies with high defect densities, which hampered definitive characterization and investigation of ligand functionalization effect. Thus, conclusive experimental, spectroscopic, and theoretical investigations are further needed to examine the supportive role of 2H‐MoS_2_ basal plane ligand functionalization to enhance electrocatalytic activity.

Herein, we demonstrate an all‐inclusive extrinsic morphological and intrinsic molecular topological reconstruction of heteroatom doped and ligand functionalized molybdenum disulfide exclusively from bimetallic (Co, Zn) organic frameworks (BMOFs). Specifically, favorable dynamic framework and weak intramolecular bonds of the BMOFs are exploited as structure‐directing features to spontaneously mobilize MoS_2_‐based molecular reconstruction. The molecular topology reassembly successfully alters the electronic structure of MoS_2_ through a new heteroatomic coordination (Zn doping) and ligand functionalization (CoS_2_ZnN) of the basal plane. A distinct HER activity is achieved for the zinc–nitrogen coordinated cobalt–molybdenum disulfide (MoS–CoS–Zn) compared with pristine molybdenum disulfide (MoS) and cobalt–molybdenum disulfide (MoS–CoS) hybrid. Comprehensive experimental spectroscopic studies and computational density functional theory (DFT) calculations reveal that electronic modulation of the active sites can be realized by efficient charge injection from the rationally designed electron‐donating functional ligand and heteroatomic coordination to the passive MoS_2_ basal planes. Moreover, considering the cooperative electrocatalytic activity tuning from i) heteroatom doping and ii) surface functionalization, the effect of each was meticulously investigated. To this end, we propose a structure‐directing metal organic framework (MOF) to coordinate functional metal centers that stimulate catalytic activity of the passive in‐plane basal MoS_2_ domains, to be comparable to the benchmark noble metal catalysts.

The strategy for the synthesis of zinc–nitrogen coordinated cobalt–molybdenum disulfide (MoS–CoS–Zn) is schematically illustrated in **Figure** **1**a and Figure S1 (Supporting Information). First, bimetallic organic framework (Co_8_Zn_1_‐MOF) (Figure 1a‐(ii)), composed of cobalt and zinc metals bridged by methyl imidazole ligands, is synthesized by solution route via partial substitution of zinc atoms for cobalt atoms in monometallic organic framework (Co‐MOF) (Figure 1a‐(i)). Co_8_Zn_1_‐MOF is then successively sulfurized to CoS–Zn and transformed into MoS–CoS–Zn nanostructure (Figure 1a‐(iii)) by two‐step solvothermal transformation, which is referred as chemical transformation in Figure 1a‐(iii). In this strategy, the Co_8_Zn_1_‐MOF is purposely exploited as a bifunctional structure‐directing precursor template to tailor the morphology and intrinsic properties of the end‐product. Figure 1b presents the typical field‐emission scanning electron microscope (SEM) image of the structure‐directing bimetallic Co_8_Zn_1_‐MOF templates. They are solid rhombododecahedral nanostructures with an average size of ≈400 nm (Figure S2, Supporting Information), possessing a Zn to Co molar ratio of 0.3 (Table S1, Supporting Information). X‐ray diffraction (XRD) pattern of Co_8_Zn_1_‐MOF (Figure S3, Supporting Information) is in a good agreement with simulated and experimental XRD patterns of the sodalite Co–MOF (ZIF‐67).^26^ Moreover, molar ratio of Zn/Co in the BMOF templates can also be tuned strategically without compromising the morphological merits (Figure S4 and Table S1, Supporting Information), endowing the possibility to control selective metal substitution with intact framework owing to the same coordination number and similar ionic radii of Zn (0.74 Å) and Co (0.72 Å). After sulfurization (Figure S5, Supporting Information) and transformation of the sulfurized MOFs into zinc–nitrogen coordinated cobalt–molybdenum disulfide (MoS–CoS–Zn), 3D nanospheric flower‐like structures composed of abundant 2D nanosheets are obtained as shown in Figure 1c. Low‐magnification transmission electron microscopy (TEM) images in Figure 1d,e clearly show that thin nanosheets surround the MoS–CoS–Zn structure. Similarly, one‐pot synthesis strategy is also carried out to obtain Zn‐coordinated MoS–CoS with similar chemical composition (Figure S1b, Supporting Information). Compared to MOF‐directed route, the Zn‐coordinated cobalt–molybdenum disulfide structures prepared via one‐pot reaction (MoS–CoS–Zn‐1pot) intensively aggregate during the reaction and eventually transform into nonuniform and large‐sized microspheres due to spontaneous bonding of cobalt and molybdenum ions with S^2−^ released from thiourea, triggered by the high sulfur concentrated‐high temperature reaction environment (Figures S6 and S7, Supporting Information). This suggests that BMOF acts as a useful template and metallic source to guide the growth of molybdenum disulfide nanosheets with superb morphological dispersity under the same condition. The BMOF template is first gradually dissolved to react with S^2−^ to form the hollow CoS–Zn via Kirkendall effect (Figure S5, Supporting Information), which is then used to host the secondary reaction of the molybdenum and S^2−^ at the rhombododecahedral surface (Figure S8, Supporting Information) to form MoS–CoS–Zn.^27^, ^28^ Compositional properties of MoS–CoS–Zn studied by TEM‐energy dispersive X‐ray spectroscopy (TEM‐EDX) confirm the presence and homogeneous distribution of the Mo, S, Co, Zn, as well as N due to nitrogen content of the MOF linker (Figure 1f–l; Table S2, Supporting Information). Besides, high‐resolution TEM (HRTEM) (Figure 1m) used to examine the nanostructured details suggests a layered structure with an interplanar spacing of 0.65 nm, which is consistent with the d‐spacing of (002) plane of hexagonal MoS_2_. In addition, the measured d‐spacing of 0.245 nm, corresponding to (210) plane of CoS_2_, confirms the coexistence of molybdenum and cobalt disulfide in the hybrid structure.^29^

**Figure 1:**
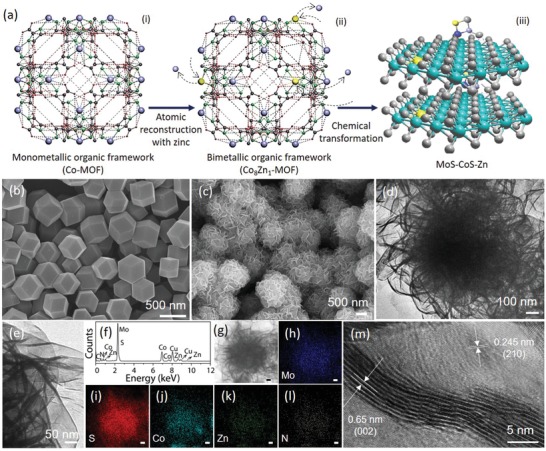
Schematic summary of the synthesis procedure and morphology analysis. a) Successive transformation of (iii) zinc–nitrogen coordinated cobalt–molybdenum disulfide from (i) monometallic organic framework (Co‐MOF). Arrows represent the key processes involved. a)‐(ii) shows the crystal structure of bimetallic organic framework (Co_8_Zn_1_‐MOF). In the MOFs ((i) and (ii)), purple, yellow, green, dark grey, and red represent Co, Zn, N, C, and H, respectively. In the MoS–CoS–Zn crystal structure ((iii)), green, grey, yellow, dark blue, and purple represent Mo, S, Zn, N, and Co atoms, respectively. b) SEM image of the bimetallic organic framework (Co_8_Zn_1_‐MOF), which is structurally presented in (a)‐(ii). c) SEM and d,e) TEM images, and f) TEM‐EDX spectrum of MoS–CoS–Zn. g) STEM image and corresponding elemental mapping images showing the distribution of h) Mo, i) S, j) Co, k) Zn, and l) N in MoS–CoS–Zn. The scale bar shows 100 nm. m) HRTEM image of the MoS–CoS–Zn.

**Figure** **2**a presents the XRD patterns of MoS_2_ (MoS) as well as MoS–CoS and MoS–CoS–Zn, which are obtained by sulfurization of monometallic Co‐MOF and bimetallic Co_8_Zn_1_‐MOF in molybdenum rich reaction environment, respectively. The XRD pattern of the MoS (Figure S9, Supporting Information) exhibits a strong diffraction peak at 2θ = 14.2° (002) and three other peaks with lower intensities at 33.6°(100), 39.9° (015), and 58.9°(110), which agree well with the diffraction patterns of hexagonal 2H‐phase of MoS_2_ (JCPDS card no. 37–1492).^30^ MoS–CoS hybrid (Figure S8, Supporting Information) displays additional peaks to 2H–MoS_2_ at diffraction angles of 32.6°, 36.5°, 46.6°, and 55.2° corresponding to (200), (210), (220), and (311) planes of CoS_2_ (JCPDS card no. 41–1471).^29^ Noticeably, the (002) plane of MoS–CoS hybrid is slightly shifted (0.6°) to lower angle compared with pristine 2H‐MoS_2_, indicating slight interlayer space expansion after hybridization (Figure S10, Supporting Information).^31^ Moreover, the broadening in (002) diffraction peak indicates that the MoS–CoS hybrid is thinner than the pristine MoS (≈15 S–Mo–S layers) and has an average thickness of 6.47 nm, which corresponds to ≈10 S–Mo–S layers. It should be noted that the XRD pattern of the MoS–CoS–Zn hybrid shows no remarkable change in the XRD peak positions, and no additional peaks attributable to Zn and its sulfides are observed, supporting trace amount of Zn coordination in the MoS–CoS–Zn. The nitrogen (N_2_) adsorption–desorption isotherms of MoS–CoS–Zn (Figure 2b) also show a Brunauer–Emmett–Teller specific surface area of 88 m^2^ g^−1^, which is higher than that of the MoS–CoS and MoS–CoS–Zn‐1pot (Figure S11, Supporting Information).

**Figure 2:**
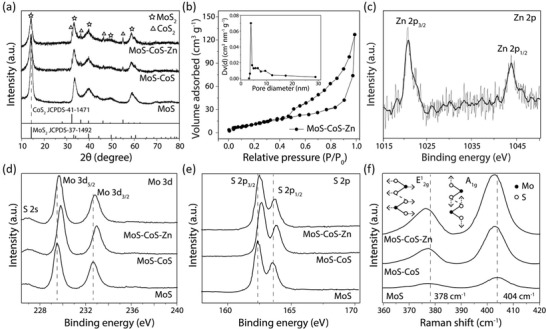
Spectroscopic characterizations of MoS, MoS–CoS, and MoS–CoS–Zn. a) X‐ray diffraction patterns of MoS, MoS–CoS, and MoS–CoS–Zn. Dotted line shows the first XRD peak position of MoS. b) N_2_ adsorption–desorption isotherm of MoS–CoS–Zn. Inset: the corresponding pore size distribution. c) High‐resolution Zn 2p spectrum of MoS–CoS–Zn. High‐resolution d) Mo 3d and e) S 2p XPS spectra of MoS, MoS–CoS, and MoS–CoS–Zn. Dotted lines in (d) and (e) show the original XPS peak positions of MoS. f) Raman spectra of MoS, MoS–CoS, and MoS–CoS–Zn. Schematic illustration presents the atomic displacements in E^1^_2g_ and A_1g_ vibrational modes, and the dotted lines indicate the corresponding peak positions of MoS.

Chemical composition and valence state of the elements were investigated by X‐ray photoemission spectroscopy (XPS). XPS spectrum of Zn 2p at 1021.5 and 1044.8 eV (Figure 2c) confirms successful Zn insertion into MoS–CoS. In the Mo 3d XPS spectrum (Figure 2d) of MoS, two peaks observed at 229.5 and 232.7 eV reflect Mo 3d_5/2_ and Mo 3d_3/2_ orbitals, respectively, indicating that Mo is in 4+ oxidation state.^32^ In a close proximity, the small peak detected at 226.7 eV is ascribed to S 2s orbital. Nonetheless, in the MoS–CoS XPS spectrum, Mo 3d and S 2s binding energies are shifted compared with MoS, suggesting interactions between MoS_2_ and CoS_2_ in MoS–CoS hybrid. Moreover, the S 2s spectra are deconvoluted into two peaks with binding energies of 226.9 and 227.5 eV, which correspond to the binding energies of S atoms in molybdenum and cobalt disulfide, respectively (Figure S12, Supporting Information). After Zn insertion into MoS–CoS, the Mo 3d_5/2_ (229.7 eV), Mo 3d_3/2_ (232.8 eV), and S 2s (226.6 and 227.1 eV) binding energies in MoS–CoS–Zn are obviously red shifted to lower binding energies in contrast to MoS–CoS hybrid. The red‐shift signifies enriched electron density around Mo, which could be ascribed to electron transfer from the coordinated Zn driven by electronegativity difference between Mo (2.16) and Zn (1.65) atoms.^33^, ^34^ Similarly, S 2p_3/2_ (162.5 eV) and S 2p_1/2_ (163.6 eV) peaks of MoS_2_ in MoS–CoS–Zn are detected at lower binding energies than those in MoS–CoS (Figure 2e), and S 2p peaks of CoS_2_ are located at higher binding energies in MoS–CoS–Zn (Figure S13, Supporting Information), further suggesting electron density localization toward Mo. This phenomenon was also confirmed by Raman spectroscopy analysis. Raman spectra of MoS, MoS–CoS, and MoS–CoS–Zn (Figure 2f) exhibit two distinct peaks at ≈378 and 404 cm^−1^, corresponding to the in‐plane (E^1^_2g_) and out‐of‐plane (A_1g_) Mo–S phonon modes, respectively. The stepwise red‐shift of the Raman peaks in MoS–CoS and MoS–CoS–Zn relative to MoS reveals softened Mo–S modes and lessened vibrational frequency.

To study the role of zinc‐coordination on electrocatalytic HER performance, activities of the electrocatalysts were assessed using a standard three‐electrode system in 0.5 m H_2_SO_4_ electrolyte by homogeneously depositing 0.25 mg cm^−2^ of catalyst slurry onto a glassy‐carbon electrode. The results are presented in **Figure** **3**, Figure S14, and Table S3 (Supporting Information). Linear sweep voltammetry (LSV) is employed at a scan rate of 5 mV s^−1^ to obtain polarization curves of the CoS, MoS, MoS–CoS, MoS–CoS–Zn, and Pt/C (Figure 3a). CoS exhibits enhanced electrocatalytic activity toward HER at overpotentials only higher than −0.35 V (vs a reversible hydrogen electrode (RHE)), and the cathodic current density cannot reach −10 mA cm^−2^ even if the applied potential is higher than −0.40 V (vs RHE). Although MoS requires a lower overpotential (373 mV) to drive the same current density (−10 mA cm^−2^), it shows rather slow cathodic current density response against increased potential. The HER polarization curve recorded on the hybrid MoS–CoS presents an overpotential of 134 mV at −10 mA cm^−2^, exceeding the catalytic activity of the pristine CoS and MoS. Impressively, the MoS–CoS–Zn shows a rapid rise in cathodic current density when the potential turns more negative, similar to Pt/C, and exhibits a superior activity with an overpotential requirement of 72.6 mV to reach −10 mA cm^−2^, which is 61.4 mV less than that of the MoS–CoS. Tafel slopes were obtained by fitting the linear region of overpotential against log j plot into Tafel equation. As shown in Figure 3b, MoS–CoS–Zn exhibits a slope of 37.6 mV dec^−1^, which outperforms the MoS–CoS (59.5 mV dec^−1^), CoS (79.9 mV dec^−1^), and MoS (114.3 mV dec^−1^), indicating that MoS–CoS–Zn provides the fastest increase of hydrogen generation rate with applied overpotential and its performance is comparable to the commercial Pt/C (30.1 mV dec^−1^). Although the Tafel slope of MoS is improved by hybridization with CoS, the value (59.5 mV dec^−1^) suggests that electrochemical desorption of hydrogen atoms is the rate‐determining reaction step, signifying the typical Volmer–Heyrovsky mechanism (H^+^ + e^−^↔H_ads_ and H^+^ + e^−^ + H_ads_ ↔ H_2_). With zinc‐coordination, however, the Tafel slope of MoS–CoS–Zn significantly decreases to 37.6 mV dec^−1^, indicating that it follows a more efficient Volmer–Tafel (H_ads_ + H_ads_ → H_2_) mechanism.^35^ In view of the improvement in electrocatalytic activity of the hybrid MoS–CoS via zinc‐coordination, influence of zinc‐coordination on the electrochemical performance of pristine CoS and MoS was also investigated (Figure 3c; Figure S15, Supporting Information). HER performance is remarkably enhanced by zinc insertion into MoS, lowering the Tafel slope (86.7 mV dec^−1^ for MoS–Zn) and overpotential (167 mV for MoS–Zn at −10 mA cm^−2^) requirement of pristine MoS.

**Figure 3:**
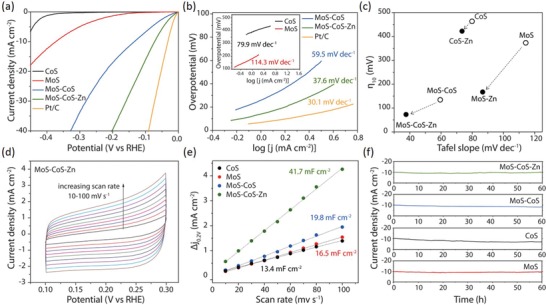
Electrochemical characterizations for the electrocatalytic HER performance of CoS, MoS, MoS–CoS, and MoS–CoS–Zn. a) HER polarization curves of CoS, MoS, MoS–CoS, MoS–CoS–Zn, and Pt/C recorded at a scan rate of 5 mV s^−1^ in 0.5 m H_2_SO_4_. b) Corresponding Tafel plots of the electrocatalysts. c) Overpotentials at a current density of −10 mA cm^−2^ and Tafel slopes comparison for pristine (CoS, MoS, and MoS–CoS) and Zn‐coordinated (CoS–Zn, MoS–Zn, and MoS–CoS–Zn) electrocatalysts. d) Cyclic voltammograms measured in a non‐Faradaic region for MoS–CoS–Zn at various scanning rates between 10 and 100 mV s^−1^. e) Estimation of double layer capacitances for CoS, MoS, MoS–CoS, and MoS–CoS–Zn using the capacitive current densities at 0.2 V (vs RHE) as a function of scan rates. f) Time dependence of current density under static overpotential showing the durability of CoS, MoS, MoS–CoS, and MoS–CoS–Zn catalysts over 60 h.

To reveal the intrinsically available charge accumulation sites of the catalysts, electrochemically active surface area (ECSA) of each catalyst was estimated by cyclic voltammetry studies (Figure S16, Supporting Information). As shown in Figure 3d, cyclic voltammograms were obtained at non‐Faradaic regions at various scan rates to determine the double‐layer capacitance (2*C*_dl_), which is linearly proportional to the ECSA. The scan‐rate dependence of the current density difference (Δ*j* = *j*_anodic_ − *j*_cathodic_ at 0.2 V vs RHE) is shown in Figure 3e. MoS–CoS–Zn possesses the highest 2*C*_dl_ of 41.7 mF cm^−2^, surpassing that of CoS (13.4 mF cm^−2^), MoS (16.5 mF cm^−2^), and MoS–CoS (19.8 mF cm^−2^), thus indicating rich active surface sites in MoS–CoS–Zn. Moreover, the ECSA values were used to normalize the measured current densities of electrocatalysts to examine their intrinsic activities (Figure S17, Supporting Information). MoS–CoS–Zn still exhibits the highest normalized current density among all, implying that HER activity improvement is associated not only with the high electrochemically active surface area but also with the high intrinsic activity of the catalytic sites in MoS–CoS–Zn. The intrinsic activity of each electrocatalyst was further studied by means of turnover frequencies (TOFs) (Figure S18, Supporting Information). It was derived from the LSV curves for HER assuming that all the atomic sites are catalytically active, signifying the lower limit of TOF.^36^ H_2_ TOF of MoS–CoS–Zn is 2.0–4.0 times larger than the values obtained for MoS and MoS–CoS at the same overpotential (150 mV), respectively. Furthermore, electrochemical impedance spectroscopy (EIS) measurements were also performed on CoS, MoS, MoS–CoS, and MoS–CoS–Zn to shed light on the high electrochemical activity of the MoS–CoS–Zn catalyst. The semi‐circles observed in the Nyquist plot (Figure S19, Supporting Information) reflect the charge transfer resistance. The Nyquist plots clearly reveal that MoS–CoS–Zn possesses a substantially smaller charge transfer resistance relative to CoS, MoS, and MoS–CoS. The EIS analysis, in conjunction with the ECSA, TOF, and normalized current density studies, corroborates that zinc coordination increases the catalytically active sites and ensures facile electron transfer; thereby offering high electrochemical activity. Besides the good catalytic activity, long‐term stability is an important requirement for practical applications. The endurance of the electrocatalysts was assessed by carrying out chronoamperometric measurements under constant applied potentials, with an initial current density of −10 mA cm^−2^, for 60 h. As shown in Figure 3f, the current density of CoS decreases gradually, while MoS shows negligible degradation over 60 h successive operation. Hybrid MoS–CoS catalyst exhibits small activity loss, which is inferior to MoS, but superior to CoS, implying the synergistic interaction. Notably, similar to the MoS–CoS, the current density of MoS–CoS–Zn generally remains stable for 60 h. Furthermore, crystal structure, elemental composition, and morphology of the MoS–CoS–Zn after 60 h continuous HER operation were investigated using SEM, TEM, XRD, and EDX characterizations (Figure S20, Supporting Information). Notably, the post‐HER MoS–CoS–Zn still retains initial morphology and crystal phase, corroborating its high stability.

Other than acidic electrolyte, HER performance of the MoS–CoS–Zn was also tested in alkaline (1 m KOH) and neutral (1 m phosphate buffer solution (PBS)) media to evaluate the universality of the MoS–CoS–Zn at different pH values. Figure S21 (Supporting Information) shows the HER polarization curves of MoS–CoS–Zn tested in 0.5 m H_2_SO_4_, 1 m KOH, and 1 m PBS electrolytes. Intriguingly, MoS–CoS–Zn exhibits remarkable electrocatalytic activity for hydrogen generation in both alkaline and neutral media as well as the acidic medium. The polarization curves demonstrate that the MoS–CoS–Zn requires overpotentials of 72.6, 85.4, and 116.2 mV to reach −10 mA cm^−2^ when tested in 0.5 m H_2_SO_4_, 1 m KOH, and 1 m PBS electrolytes, respectively. Moreover, MoS–CoS–Zn exhibits small Tafel slopes in both alkaline (49.1 mV dec^−1^) and neutral media (58.6 mV dec^−1^), signifying that MoS–CoS–Zn is an efficient, pH‐universal electrocatalyst. The overpotentials and Tafel slopes of the MoS–CoS–Zn indicate that it exhibits comparable or higher electrocatalytic activity than the recently reported MoS_2_‐based catalysts (Table S3, Supporting Information).

The increase in HER performance with zinc coordination could be ascribed to the electronic environment modification as supported by XPS and Raman spectroscopy. It was further elucidated by examining the energy requirements related to the occurrence hydrogen evolution reaction. The activation energy barrier of an electrocatalyst can be altered by modifying the work function and electronic band structure.^37^ Here, they were determined for MoS–CoS and MoS–CoS–Zn by carrying out ultraviolet photoelectron spectroscopy (UPS) (**Figure** **4**a–c). As presented in Figure 4a, MoS–CoS–Zn exhibits a valence band maximum value of 0.48 eV, which is much closer to the Fermi level (set to 0 eV) than 0.80 eV obtained for MoS–CoS. This signifies that reconstructed MoS–CoS–Zn surface with zinc insertion induces more metallic character with higher density of states around Fermi level as also indicated by intense characteristic valence band maximum peak in Figure S22 (Supporting Information). Furthermore, work function of MoS–CoS is found to be 5.15 eV and decreased by 0.35 eV upon insertion of zinc to 4.8 eV (Figure 4b). Decrease in work function is beneficial for liberating electrons from the electrocatalyst to the surface, thereby facilitating the hydrogen evolution reaction on the catalyst surface. Based on the UPS investigations, schematic energy diagram in Figure 4c represents the energy level orientation of MoS–CoS and MoS–CoS–Zn with respect to the water dissociation potential.^38^ Compared with MoS–CoS, lower energy is required to liberate the electrons from MoS–CoS–Zn, which, from the thermodynamic viewpoint, supports the lower overpotential requirement of the zinc‐coordinated structures.

**Figure 4:**
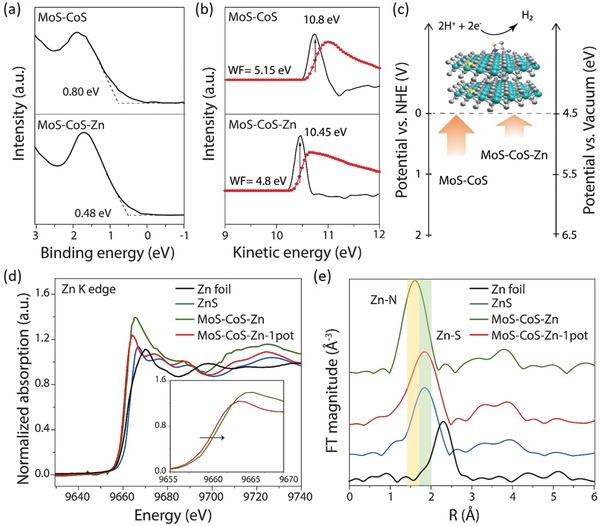
Structural and electronic properties of the MoS–CoS–Zn. a) Low binding energy edge of UPS showing the valence band maxima of MoS–CoS and MoS–CoS–Zn with respect to Fermi level. b) UPS investigations for work function. The red and black dotted lines present the data measured by the instrument and the differentiate curve, from which kinetic energy at the secondary electron edge is obtained at the peak position. Details of the calculations can be found in the Supporting Information. c) Schematic representing the energy level orientations of MoS–CoS and MoS–CoS–Zn with respect to the water dissociation potential. d) Normalized Zn K‐edge XANES spectra of MoS–CoS–Zn, MoS–CoS–Zn‐1pot, ZnS, and Zn foil. Inset: the absorption edge between 9655 and 9670 eV. e) Zn K‐edge k^2^‐weighted EXAFS spectra of MoS–CoS–Zn, MoS–CoS–Zn‐1pot, ZnS, and Zn foil. The highlighted regions show Zn–N and Zn–S coordination peak ranges.

Although heteroatom doping has been shown to trigger the electrocatalytic activity as explicitly shown in this work and recent studies,^33^, ^39^, ^40^, ^41^ the same heteroatoms with different coordination configuration could result in distinctively different performance. As presented in the polarization curve in Figure S23 (Supporting Information), MoS–CoS–Zn‐1pot exhibits inferior catalytic activity compared with MoS–CoS–Zn synthesized by MOF‐route. To reach the current density of −10 mA cm^−2^, it requires an overpotential of 128 mV, which is more than 1.7 times of that of MoS–CoS–Zn. To attain understanding on the discrete performances of these two electrocatalytic systems, atomic and local electronic structures of zinc‐coordinated cobalt–molybdenum disulfides were investigated by carrying out X‐ray absorption near‐edge structure (XANES) and extended X‐ray absorption fine structure (EXAFS) spectroscopy. Figure 4d presents the Zn K‐edge spectra of MoS–CoS–Zn and MoS–CoS–Zn‐1pot. Spectrum of zinc sulfide (ZnS) (Figure S24, Supporting Information) was also investigated for comparison. The Zn K‐edge XANES spectra show that the white line intensities of molybdenum–cobalt coordinated zinc structures (MoS–CoS–Zn and MoS–CoS–Zn‐1pot) are stronger than that of pristine ZnS, revealing higher oxidation state of Zn species in the molybdenum–cobalt coordinative environment. Specifically, Zn in MoS–CoS–Zn possesses higher peak intensity and positive shift in the absorption edge compared with MoS–CoS–Zn‐1pot (Figure 4d inset), which suggests that Zn is more positively charged, and therefore more electron transfer could occur from Zn to MoS–CoS hybrid in MoS–CoS–Zn, coinciding with the XPS spectra data.^42^ In addition, MoS–CoS–Zn exhibits a near‐edge structure different from those of ZnS and MoS–CoS–Zn‐1pot, revealing a dissimilar local coordination environment of Zn in MoS–CoS–Zn. This finding is corroborated by Fourier‐transformed EXAFS (FT‐EXAFS) spectra of MoS–CoS–Zn, MoS–CoS–Zn‐1pot, and ZnS. As shown in Figure 4e, the Zn K‐edge FT‐EXAFS spectrum of MoS–CoS–Zn exhibits significant differences compared with MoS–CoS–Zn‐1pot and ZnS. The main peak of MoS–CoS–Zn‐1pot at 1.9 Å is associated with Zn–S peak, which is also significant in the ZnS spectrum. Moreover, the secondary peak positions (Zn–Zn) of MoS–CoS–Zn‐1pot and ZnS are also similar, indicating that the atomic structures surrounding Zn in MoS–CoS–Zn‐1pot are analogous to those in ZnS. On the other hand, the main Zn K‐edge peak in MoS–CoS–Zn is located at a lower value (1.6 Å), corresponding to Zn–N interatomic distance, and the other secondary FT peaks are not similar to those of MoS–CoS–Zn‐1pot and ZnS.^43^ Zn—N bond can be also further confirmed by Zn 2p spectrum of MoS–CoS–Zn (Figure 2c), which shows two peaks at 1021.5 and 1044.8 eV, corresponding to 2p_3/2_ and 2p_1/2_ orbitals of Zn–N, respectively.^44^, ^45^, ^46^ To further reveal the electronic environment modulation, and to verify the Zn atom coordination, X‐ray absorption fine structure measurements on Co (Figure S25, Supporting Information) and Mo K‐edges (Figure S26, Supporting Information) were also carried out. The Co K‐edge XANES spectrum of MoS–CoS–Zn‐1pot (Figure S25a, Supporting Information) exhibits 1s→3d pre‐edge peak at 7710 eV, corresponding to a cobalt oxidation state of 2+.^47^ In the Co K‐edge spectrum of MoS–CoS–Zn, stronger white line intensity and more positive absorption edge position are observed. These results agree well with the observations of Zn K‐edge spectra, indicating strong electronic interactions in MoS–CoS–Zn structure. According to the analysis of the first shell (Figure S25b, Supporting Information), MoS–CoS–Zn‐1pot exhibits a Co—S distance of 1.86 Å, which agrees well with the Co—S bond length in CoS_2_ crystal structure.^48^ The Co—S coordination in MoS–CoS–Zn shifts to a lower R‐position (1.80 Å), demonstrating that the main coordination mode of Co differs compared with MoS–CoS–Zn‐1pot. As revealed by the Co K‐edge investigations, despite the similar coordination structures of cobalt atoms in MoS–CoS–Zn and MoS–CoS–Zn‐1pot, they possess different electronic structures. Figure S26a (Supporting Information) shows the Mo K‐edge spectra of MoS–CoS, MoS–CoS–Zn, and MoS–CoS‐1pot. A closer examination of the spectra (Figure S26a inset, Supporting Information) shows that zinc‐coordinated samples exhibit slight change in white line feature relative to MoS–CoS indicating electronic environment modification with small amount of zinc incorporation. FT‐EXAFS at the Mo K‐edge was studied to investigate the coordination environment around Mo atom (Figure S26b, Supporting Information). Two main peaks detected in MoS–CoS spectra at 1.9 and 2.9 Å correspond to Mo—S and Mo—Mo bonds, respectively.^49^

As revealed by the XPS, Raman, UPS, and XANES studies, Zn‐insertion into MoS–CoS structure through the MOF‐directed synthesis strategy changes the electronic structure of MoS_2_ basal plane in a noticeable way. To the basal planes of pristine 2H–MoS_2_, hydrogen adsorbs weakly with an endothermic adsorption Gibbs free energy (Δ*G*_H_) of ≈2 eV.^41^ However, adsorption strength of hydrogen on an electrocatalytic surface can be made favorable by tuning the Bader charge of atoms via changing the electronic structure.^39^, ^40^, ^41^ Depending on the spectroscopic findings, charge injection is realized from Zn centers to Mo, which could subsequently regulate the electron number on S and Mo atoms and offset energy level mismatching for favorable hydrogen adsorption, as revealed by the UPS investigations. Moreover, presence of additional electrons on N centers can also contribute for a facile hydrogen adsorption, thereby accelerating the rate of catalytic reaction.^14^ To gain further insights into the charge‐injection enhanced HER activity, systematic density functional theory calculations were carried out based on the experimental spectroscopic studies, which suggests zinc, nitrogen, and cobalt sulfide coordination to molybdenum disulfide (Figure S27, Supporting Information). We first studied the effect of in‐plane zinc‐doping on electronic properties and hydrogen adsorption behavior of MoS_2_ (MoS_2_–Zn, corresponding to MoS–Zn notation). The calculations show that the substitution of a Mo atom by a Zn atom in the MoS_2_ supercell (see Figure S28a, Supporting Information) leads to a significant charge transfer from the Zn dopant to MoS_2_, which is around 0.9*e* based on the Bader charge analysis (Figure S29, Supporting Information).^50^ As shown in **Figure** **5**a, such strong charge transfer introduces a few gap states from MoS_2_, not from the Zn dopant, suggesting an effectively improved electronic conductivity of MoS_2_ by Zn substitution. The calculated Gibbs free energies of adsorbed hydrogen show that the basal‐plane S top site (Δ*G*_H_ = −0.164 eV) and the S–S bridge site near the Zn dopant (Δ*G*_H_ = 0.132 eV) are activated toward HER (Figure 5d). Next, we studied a CoS_2_ZnN cluster on pristine MoS_2_ (denoted as CoS_2_ZnN@MoS_2_) (Figure S28b, Supporting Information). The calculated adsorption energy of −1.35 eV indicates strong adsorption of CoS_2_ZnN to MoS_2_ (Figure S29, Supporting Information). However, there is no charge transfer between the cluster and pristine MoS_2_ due to local accumulation of the electrons in between (Figure S28b, Supporting Information). We further considered the CoS_2_ZnN cluster adsorption on the Zn‐doped MoS_2_ (CoS_2_ZnN@Zn–MoS_2_, corresponding to MoS–CoS–Zn notation). Here, seven possible CoS_2_ZnN adsorption sites nearby the Zn on the MoS_2_ supercell (Figure S30, Supporting Information) were investigated to optimize the suggested CoS_2_ZnN coordination by the spectroscopic studies. The CoS_2_ZnN cluster contributes ≈0.22–0.32 *e* to MoS_2_–Zn in most of the cases, indicating that the electronic conductivity of MoS_2_ is further enhanced by strong adsorption of the cluster (Figure S29, Supporting Information). In particular, we scrutinize the most energetically stable CoS_2_ZnN adsorption case, i.e., case 4 (Figure 5b; Figures S29 and S30, Supporting Information). The calculated projected density of states (PDOS) of the adsorption case 4 is shown in Figure 5c. It is clear that more gap states are introduced when the cluster is adsorbed near the Zn dopant, confirming the further enhancement of the MoS_2_ electronic conductivity. Besides, the calculated Gibbs free energies in the CoS_2_ZnN adsorption case 4 suggest that the CoS_2_ZnN cluster can dramatically enhance the HER performance of Zn–MoS_2_ (Figure 5d). With the presence of the cluster, the HER activities of the S top sites 1 and 2 (see Figure 5b; Figure S31, Supporting Information) are further improved by one order of magnitude. As for two bridge sites, our adsorption‐energy calculations show that the hydrogen is hard to adsorb on the S–S bridge site 1 for the Volmer reaction, while the initial adsorption on S–S bridge site 2 is not stable, it evolves into a more promising hydrogen adsorption configuration (see Figure S31b, Supporting Information) with the Gibbs free energy of only 0.03 eV. It is worth noting that the Gibbs free energies of the S top site 1 and the S–S bridge site 2 are comparable to that of Pt.^51^ In summary, Zn dopants on the MoS_2_ surface can introduce active sites for HER, resulting in a Gibbs free energy requirement of ≈0.15 eV. Most importantly, the presence of the CoS_2_ZnN nearby the Zn dopant on MoS_2_ can further enhance the intrinsic activity of the MoS_2_ basal planes by optimizing the hydrogen adsorption ability, as revealed by the moderate Gibbs free energy down to 0.03 eV, which is ascribed to the improved conductivity due to remarkable charge transfer from both Zn dopants and adsorbed CoS_2_ZnN clusters to the MoS_2_ basal planes.

**Figure 5:**
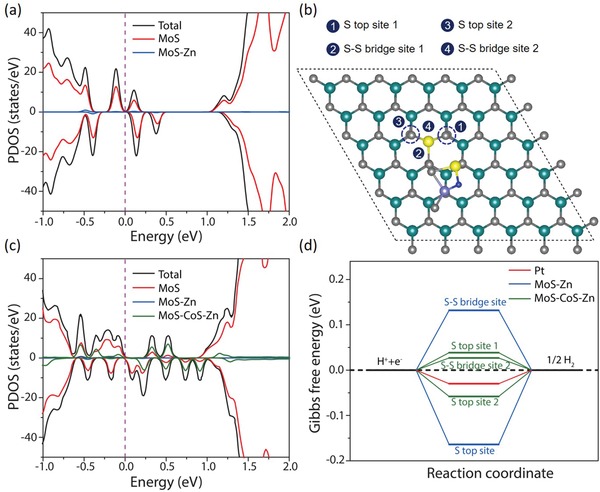
DFT studies for the effect of zinc coordination on electrocatalytic HER activity. a) Calculated PDOS of Zn–MoS_2_ (MoS–Zn), a 6 × 6 × 1 MoS_2_ supercell with one Mo atom replaced by a Zn atom. b) The top view of the CoS_2_ZnN adsorption case 4 on Zn–MoS_2_ (MoS–CoS–Zn) (see Figures S24 and S25, Supporting Information). c) Calculated PDOS of the CoS_2_ZnN@Zn–MoS_2_ (MoS–CoS–Zn) in (b). d) The calculated hydrogen adsorption Gibbs free energy diagram of Zn–MoS_2_ (MoS–Zn) and CoS_2_ZnN@Zn–MoS_2_ (MoS–CoS–Zn) along with Pt for comparison.

In summary, sulfurization of zinc–cobalt containing BMOF, a favorable dynamic metalloligands network assembled by weak intramolecular bonds, with molybdenum atoms triggered reconstruction of zinc–nitrogen coordinated cobalt–molybdenum disulfide molecular structure. Detailed experimental spectroscopic studies (XPS, UPS, Raman, XANES) and first‐principle calculations (DFT) affirmed the intrinsic molecular coordination of electron‐rich centers, suggested efficient charge injection through in‐plane inert MoS_2_ sites and confirmed lower thermodynamic potential requirement of zinc–nitrogen coordinated MoS–CoS molecular structure. It was demonstrated that the basal plane of the 2H phase can be made as catalytically active as the edges by in situ creation of intact electron‐rich centers. Notably, MoS–CoS–Zn exhibited a superior electrochemical activity toward HER with an overpotential of −72.6 mV (vs RHE) at 10 mA cm^−2^ and a Tafel slope of 37.6 mV dec^−1^. This work is believed to open up new opportunities for development of highly active molybdenum disulfide HER electrocatalysts via in situ structural design and functionalization.

## Conflict of Interest

The authors declare no conflict of interest.

## Supporting information

Supplementary
