# Supplementary material for: Stimulated Electrocatalytic Hydrogen Evolution Activity of MOF‐Derived MoS2 Basal Domains via Charge Injection through Surface Functionalization and Heteroatom Doping
Source: Adv Sci (Weinh). 2019 May 29;6(15):1900140. doi: 10.1002/advs.201900140 (PMC6685470; doi:10.1002/advs.201900140)
Supplement: Supplementary file 1 — Supplementary [file ADVS-6-1900140-s001.pdf]

Copyright WILEY-VCH Verlag GmbH & Co. KGaA, 69469 Weinheim, Germany, 2019.

ADVANCED SCIENCE
Open Access

Supporting Information

for *Adv. Sci.,* DOI: 10.1002/advs.201900140

Stimulated Electrocatalytic Hydrogen Evolution Activity of MOF-Derived MoS_2 Basal Domains via Charge Injection through Surface Functionalization and Heteroatom Doping

*Gamze Yilmaz, Tong Yang, Yonghua Du, Xiaojiang Yu, Yuan Ping Feng, Lei Shen, and Ghim Wei Ho**

Supporting Information for

Stimulated Electrocatalytic Hydrogen Evolution Activity of MOF-derived MoS_2 Basal Domains via Charge Injection through Surface Functionalization and Heteroatom Doping

*Gamze Yilmaz[1], Tong Yang[2], Yonghua Du[3], Xiaojiang Yu[4], Yuan Ping Feng[2], Lei Shen[5] and Ghim Wei Ho[1,6]**

[1]Department of Electrical and Computer Engineering, National University of Singapore, 4 Engineering Drive 3, 117583, Singapore. [2]Department of Physics, National University of Singapore, 117551, Singapore. [3]Institute of Chemical and Engineering Sciences, A*STAR (Agency for Science, Technology and Research), 1 Pesek Road, Jurong Island, 627833, Singapore. [4]Singapore Synchrotron Light Source, National University of Singapore, 5 Research Link, 117603, Singapore. [5]Department of Mechanical Engineering, National University of Singapore, 117575, Singapore. [6]Institute of Materials Research and Engineering, A*STAR (Agency for Science, Technology and Research), 2 Fusionopolis Way, Innovis, 138634, Singapore.

Methods

Materials synthesis.

Synthesis of Co_xZn_y-MOF. 3 mmol of cobalt nitrate hexahydrate ($Co(NO_3)_2.6H_2O$) and zinc nitrate hexahydrate ($Zn(NO_3)_2.6H_2O$) in total were dissolved in 50 mL absolute methanol to prepare solution A. For example, in a typical experiment for synthesis of Co_8Zn_1-MOF, 2.67 mmol of $Co(NO_3)_2.6H_2O$ and 0.33 mmol of $Zn(NO_3)_2.6H_2O$ were dissolved in 50 mL absolute methanol to prepare solution A. Then, in a separate container, 0.73 g 2-Methylimidazole was added in 50 mL absolute methanol to form solution B. Solution B was then added into Solution A, stirred for 5 min, and aged for precipitation for 24 h. After aging, the products were collected by centrifugation, washed with ethanol for at least two times, and dried in an oven at 60°C for 12 h.

Synthesis of Co-MOF. The synthetic procedure of Co-MOF is the same with that of Co_xZn_y-MOF, except the addition of $Zn(NO_3)_2.6H_2O$.

Synthesis of MoS. 0.1 g of sodium molybdate dihydrate ($Na_2MoO_4.2H_2O$) and 0.2 g of thiourea (TU) were dissolved in 25 mL of deionized (DI) water. The obtained solution was then transferred into a 50 ml Teflon-lined autoclave, which was sealed and maintained at 220℃ for 24 h. After cooling down room temperature, MoS_2 was collected by centrifugation, washed by DI water for three times, and the dried in an oven at 60℃ for 12 h.

Synthesis of ZnS. 0.1 g of zinc acetate ($ZnAc_2$) and 1.5 g of polyvinylpyrrolidone (PVP) were dissolved in 10 ml DI water and stirred for 6 h at room temperature. Then, 0.04 g of thioacetamide (TAA) was dissolved in 10 mL DI water in a separate container. The TAA solution was added into $ZnAc_2$ solution. After that, the obtained solution was transferred into a 25 ml Teflon-lined autoclave, which was sealed and maintained at 220℃ for 24 h. After cooling down room temperature, ZnS was collected by centrifugation, washed by DI water for three times, and the dried in an oven at 60℃ for 12 h.

Synthesis of CoS and CoS-Zn. 30 mg of Co-MOF and $Co_{15}Zn_1$-MOF, and 40 mg of TAA were dissolved in 40 mL of absolute ethanol to prepare the reaction solutions of CoS and CoS-Zn, respectively. The obtained solution was then transferred into a 50 ml Teflon-lined autoclave, which was sealed and maintained at 100℃ for 1 h. After cooling down room temperature, CoS and CoS-Zn were collected by centrifugation, washed by ethanol for three times, and the dried in an oven at 60℃ for 12 h.

Synthesis of MoS-CoS and MoS-CoS-Zn. As-synthesized CoS and CoS-Zn (prepared using Co_8Zn_1-MOF), 0.1 g of $Na_2MoO_4.2H_2O$ and 0.2 g of TU were dissolved in 25 mL of DI water to prepare the reaction solutions of MoS-CoS and MoS-CoS-Zn, respectively. The obtained solution was then transferred into a 50 ml Teflon-lined autoclave, which was sealed and maintained at 220℃ for 24 h. After cooling down

room temperature, MoS-CoS or MoS-CoS-Zn were collected by centrifugation, washed by DI water for three times, and the dried in an oven at 60℃ for 12 h.

Synthesis of MoS-CoS-Zn-1pot. 0.05 g of $Na_2MoO_4.2H_2O$, 0.04 g of $Co(NO_3)_2.6H_2O$ and 0.01 g of $Zn(NO_3)_2.6H_2O$ and 0.2 g of TU were dissolved in 25 mL of DI water. The obtained solution was then transferred into a 50 ml Teflon-lined autoclave, which was sealed and maintained at 220℃ for 24 h. After cooling down room temperature, the product was collected by centrifugation, washed by DI water for three times, and the dried in an oven at 60℃ for 12 h.

Materials characterization. Field emission-scanning electron microscopy (FESEM) was performed on a JEOL FEG JSM-7001F, equipped with an Oxford/INCA EDS, to study the morphology of the materials. Transmission electron microscopic (TEM) and high-resolution transmission electron microscopic (HRTEM) images were recorded by a JEOL JEM-2010 F microscope at an acceleration voltage of 300 kV. The elemental mapping of the materials was performed by energy-dispersive X-ray spectroscope (EDX) attached to the JEOL JEM-2010 F TEM instrument. The crystal structure was obtained by X-ray powder diffraction (XRD) by collecting the patterns using a diffractometer (GADDS XRD system, Bruker AXS) equipped with a CuKα radiation source (λ= 1.54 Å). X-ray photoelectron spectroscopy (XPS) measurements were performed on a PHI Quantera x-ray photoelectron spectrometer with a monochromated Al Kα radiation. Raman spectrum was obtained using a 532 nm excitation laser with a WITec Raman instrument. Nitrogen adsorption-desorption isotherms were obtained at 77.3 K using the (Quantachrome) NOVA-1200 System and pore size distribution was calculated by BJH method based on desorption isotherm.

The UPS measurements were carried out using the SINS beamline of Singapore Synchrotron Light Source equipped with a VG Scienta R4000 analyser.[1] The work-functions (WFs) were measured with an excited

photon energy of 60 eV and the sample is biased at -10 V. The following formula was used to calculate work function:

$$WF = KE + bias + \Phi_I$$

where, Φ_I is the instruments work function ($\Phi_I = 4.35\ eV$), KE is the kinetic energy at the secondary electron edge, which is at the peak position of the differentiate curve.

Zn and Co K-edge X-ray absorption fine structure (XAFS) data was recorded using XAFCA beamline at the Singapore Synchrotron Light Source (SSLS).[2] Zn and Co foil were applied for the energy correction of the Zn and Co K-edge spectra, respectively. The Mo K-edge XAFS data was collected at 1W1B of Beijing Synchrotron Radiation Facility (BSRF) under transmission mode. Mo foil was applied for the energy correction.

Electrode preparation and electrochemical measurements.

HER tests. The working electrode was prepared by mixing 5 mg of active material with 0.95 mL ethanol and 0.05 mL Nafion® 117 solution (5 wt%). The mixture was sonicated for 30 min to prepare a homogeneous ink. Then, 6.3 µl of the ink dispersion was loaded onto the glassy carbon (GC) disk of 4 mm in diameter, leading to a catalyst loading of 0.25 mg cm^{-2}. Finally, it was dried at room temperature. All electrochemical measurements were conducted on a CHI 660E electrochemical workstation with a standard three-electrode set-up using the as-synthesized materials as working electrode, saturated calomel electrode (SCE) as reference electrode and carbon rod as counter electrode in 0.5 M aqueous H_2SO_4. Linear sweep voltammetry (LSV) was recorded at a scan rate of 5 mVs^{-1} to obtain the polarization curves and Tafel plots. Chronoamperometric i-t profile data was collected for 60 h under constant overpotential. Electrochemical impedance spectroscopy (EIS) tests were performed with a frequency loop from 100 kHz to 0.01 Hz by applying a sine wave with an amplitude of 5 mV at an overpotential of 0.1 V. Before HER tests, each working electrode was cycled 20 times at 50 mVs^{-1} until getting a

reproducible CV. Conversion of SCE potential to RHE potentials was done according to the following equation: E(RHE)=E(SCE)+0.2412+0.05916×pH.

Theoretical methodology. First-principles calculations were performed based on spin-polarized density functional theory (DFT) using the generalized gradient approximation of Perdew, Beurke and Ernzerhof (GGA-PBE) for the exchange-correlation functional,[3] implemented in the Vienna *ab-initio* Simulation Package (VASP).[4-5] The ion-electron interaction was described by the projector augmented-wave (PAW) method.[6] The wave functions were expanded in a plane-wave basis with a cutoff energy of 550 eV. The first Brillouin zone was sampled with a gamma-centered 15×15×1 k-mesh for the MoS_2 unit cell. The electronic and ionic convergence criteria were set to 10^{-4} eV and 0.01 eV/Å for energy and force, respectively. A vacuum of around 20 Å was used on the surface of MoS_2 to eliminate the interaction between periodic images. A large 6×6×1 supercell was adopted to investigate the effects of doping of Zn atoms and the adsorption of CoS_2ZnN clusters on MoS_2. Both the long-range van der Waals interaction[7-8] and the dipole correction[9-10] were included in our calculations. The optimized lattice constant of the MoS_2 unit cell is 3.185 Å, in agreement with the reported result[11].

Gibbs free energy for adsorbed hydrogen, ΔG_{H}, is a good descriptor to evaluate the catalytic activity towards HER,[12-13] which is defined as $\Delta G_{\mathrm{H}} = \Delta E_{\mathrm{H}} + \Delta E_{\mathrm{ZPE}} - T\Delta S_{\mathrm{H}}$, where ΔE_{H} is the hydrogen adsorption energy, defined as $\Delta E_{\mathrm{H}} = E(H + cat) - E(cat) - 1/2\,E(H_2)$, where $E(H + cat), E(cat)$ and $E(H_2)$ are the energies of the catalyst with a hydrogen atom adsorbed, the pristine catalyst and the gas-phase H_2 molecule, respectively. In this study, the catalyst is a Zn doped MoS_2, and Zn doped MoS_2 with an adsorbed CoS_2ZnN cluster. ΔE_{ZPE} and ΔS_{H} represent the change in the zero-point energy (ZPE) and the change in the entropy of hydrogen upon the hydrogen adsorption. Since the entropy of the adsorbed H is very small, $T\Delta S_{\mathrm{H}} \approx -TS_{\mathrm{H}}{}^{0} = -0.202$ eV at $T = 298$ K.[14] The optimal HER activity can be

achieved as ΔG_{H} goes to zero, where both the hydrogen adsorption and the subsequent desorption can be facilitated.[12-13]

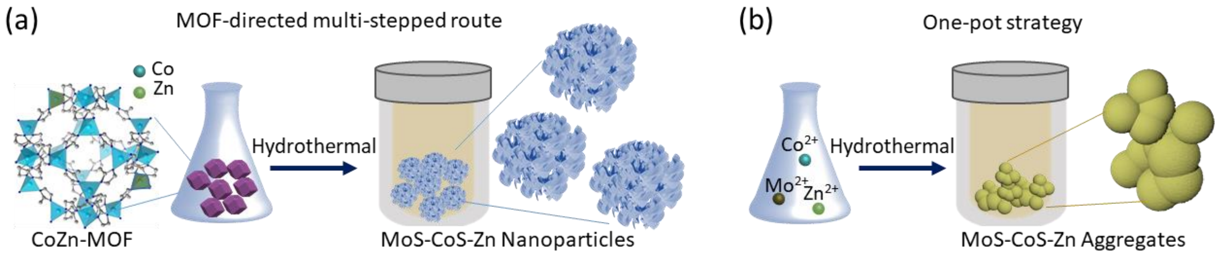

Figure S1. Schematic illustration for the formation process of MoS-CoS-Zn. (a) MOF-directed route and (b) one-pot strategy. CoZn-metal organic framework (MOF) was synthesized by incorporating zinc atoms into monometallic cobalt-based zeolitic imidazolate framework (ZIF)-67. CoZn-MOF was then used to prepare zinc-coordinated molybdenum-cobalt sulfide structures. As illustrated in Figure S1a, monodispersed nanoflower-like structures are obtained by MOF-directed route. On the other hand, direct synthesis of zinc-coordinated molybdenum-cobalt sulfide structures following the one-pot strategy results in formation of agglomerate nanostructures.

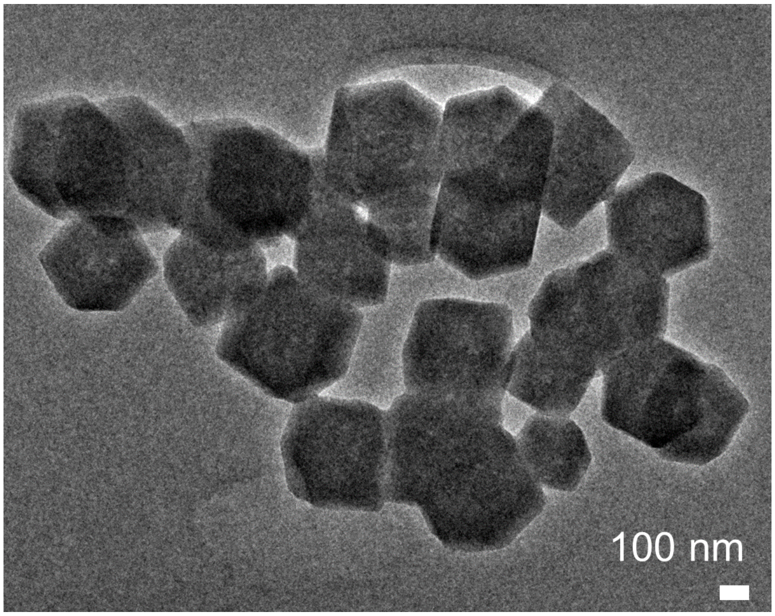

Figure S2. TEM image of Co_8Zn_1-MOF. The structure-directing bimetallic Co_8Zn_1-MOF templates are solid rhombododecahedral nanostructures with an average size of ~400 nm.

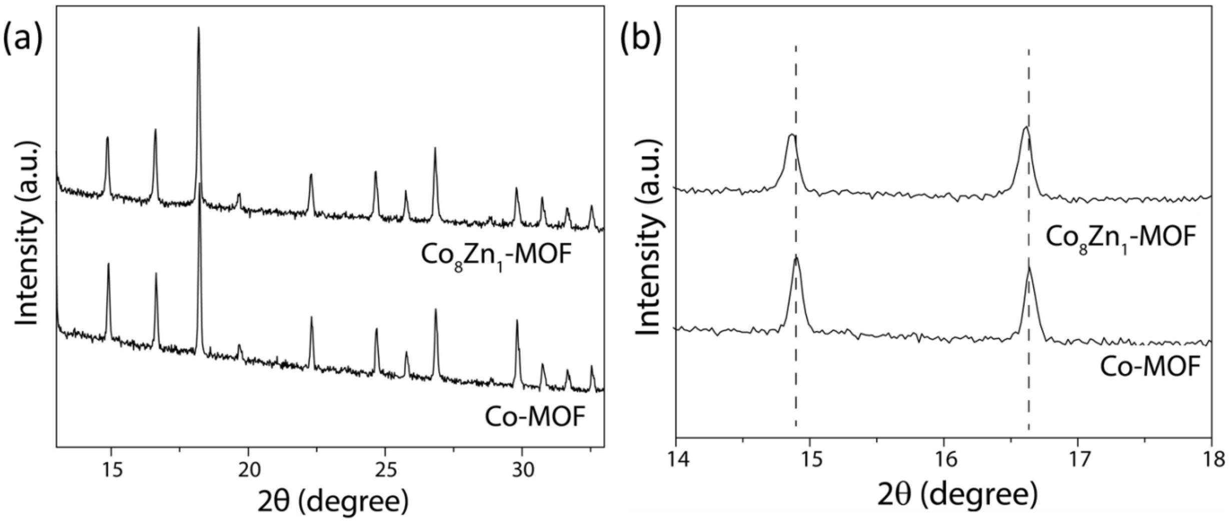

Figure S3. XRD investigations of the Co_8Zn_1-MOF. (a) XRD patterns of Co_8Zn_1-MOF and Co-MOF. (b) Zoomed-in view of the XRD patterns. XRD peaks of the Co-MOF match well with the experimental and simulated XRD patterns of ZIF-67.[15] As shown in Figure S3a, the Co_8Zn_1-MOF exhibits sharp diffraction peaks, indicating that it exhibits a highly crystalline structure. Moreover, diffraction pattern of the Co_8Zn_1-MOF is similar to that of the Co-MOF, which reveals that Co_8Zn_1-MOF retains the crystalline framework of Co-MOF. Since the ionic radii of zinc and cobalt are similar (0.74 Å and 0.72 Å, respectively) and they have the same coordination number, zinc ions can readily substitute cobalt ions. This was further supported by investigating the zoomed-in XRD patterns. Figure S3b shows that peaks of Co-MOF are slightly shifted to lower angles, confirming the substitutional constitution of slightly larger zinc atoms for cobalt atoms.

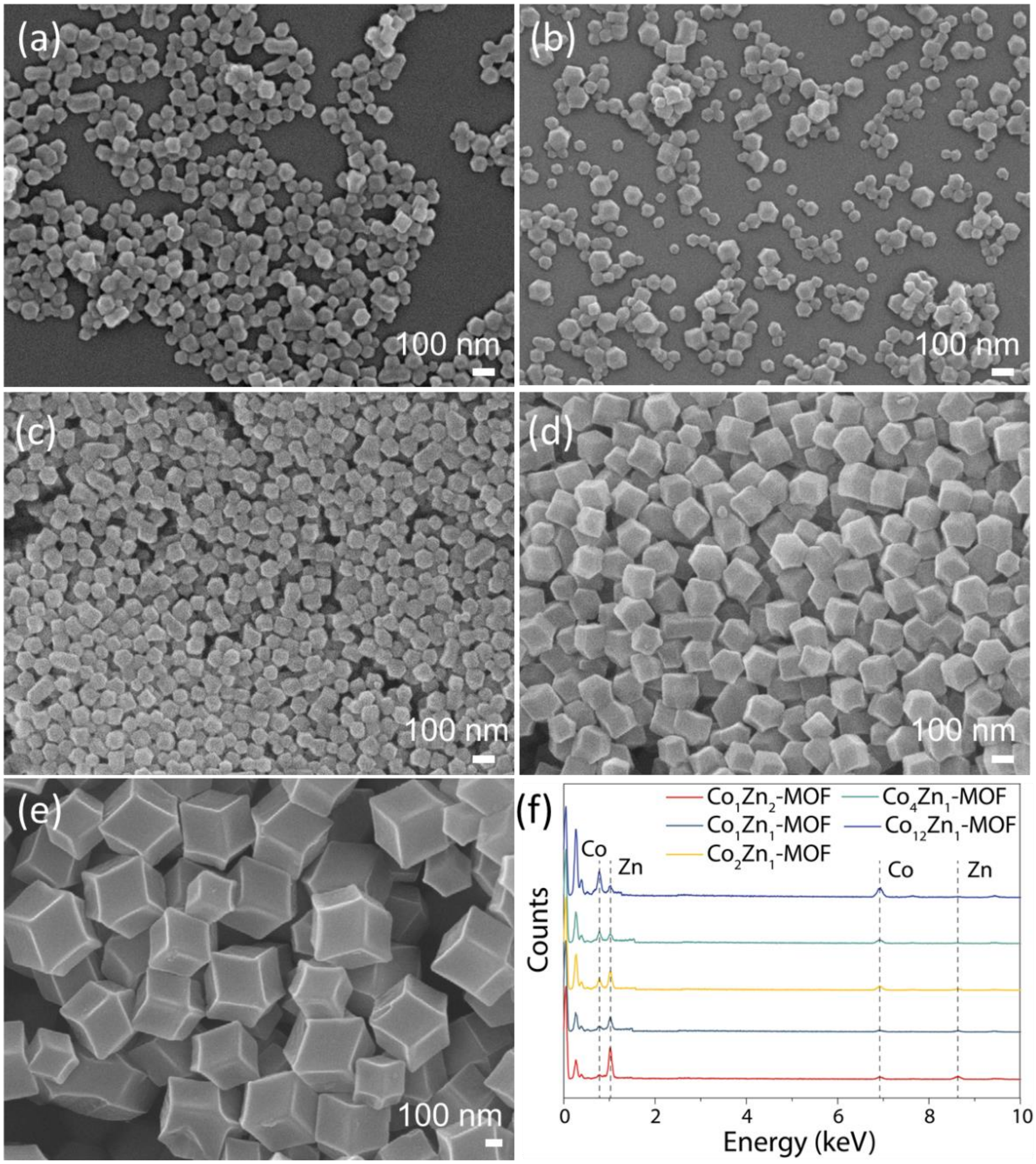

Figure S4. Morphological and chemical analysis of Co_xZn_y-MOFs. SEM images of Co_xZn_y-MOFs: (a) Co_1Zn_2-MOF, (b) Co_1Zn_1-MOF, (c) Co_2Zn_1-MOF, (d) Co_4Zn_1-MOF and (e) $Co_{12}Zn_1$-MOF. (f) SEM-EDX spectra of Co_xZn_y-MOFs. As shown in Figure S4a-e, molar ratio of Zn/Co in the bimetallic templates can be tuned strategically without compromising the morphological merits, endowing the possibility to control selective metal substitution. Figure S4f supports the successful tuning of the cobalt and zinc contents.

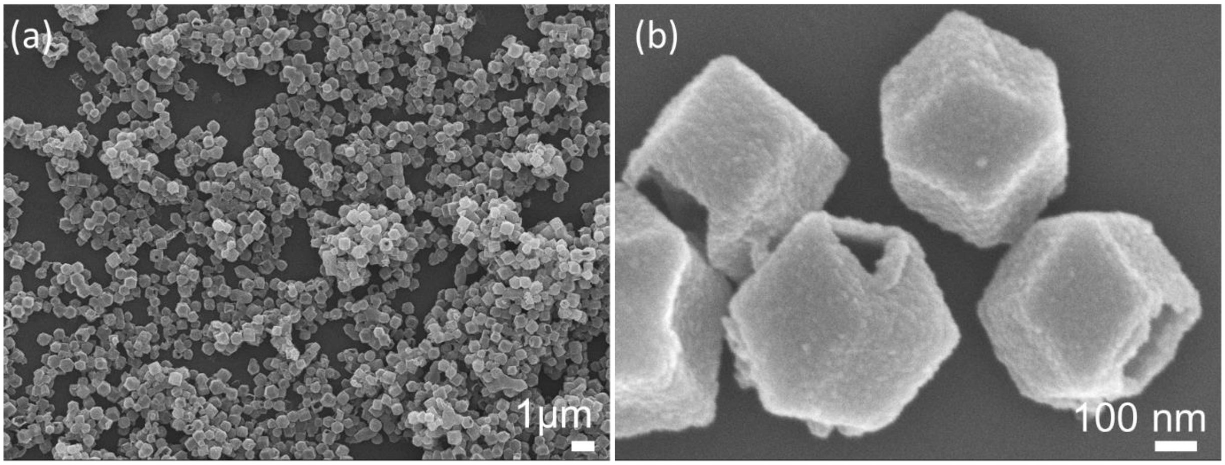

Figure S5. SEM images of sulfurized Co_8Zn_1-MOF. (a) and (b) SEM images of Co_8Zn_1-MOF at different magnifications. Uniformity of the as-synthesized structures can be discerned well by the SEM images in Figure S5a. Sulfurized Co_8Zn_1-MOF retains the morphology of initial templates even after sulfurization treatment.

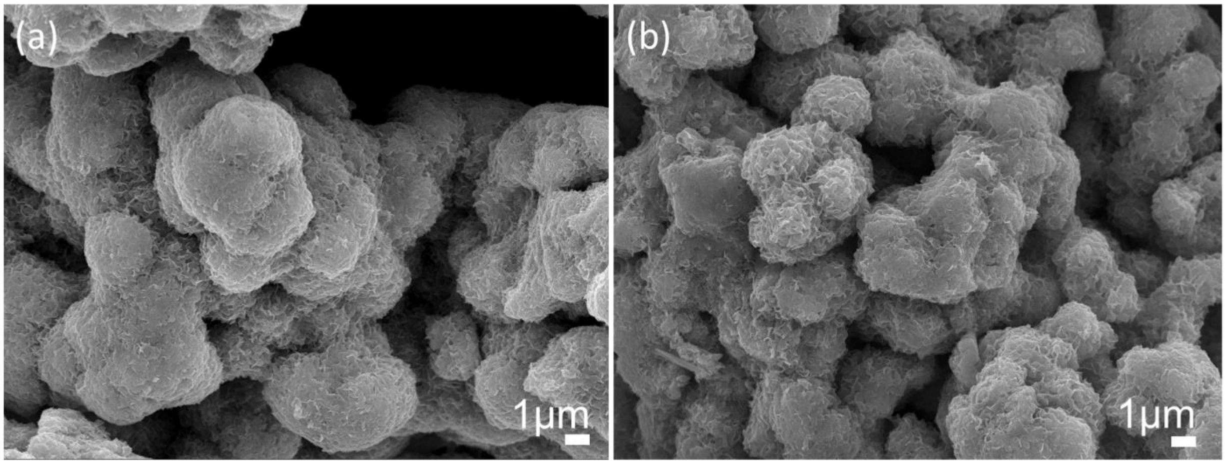

Figure S6. SEM images of the MoS-CoS-Zn-1pot. (a) and (b) SEM images of MoS-CoS-Zn-1pot taken from different areas. Direct one-pot hydrothermal reaction favors the formation of aggregate nanostructures. The precursors form non-uniform large-sized microspheres (>2 μm) as shown in Figure S6a,b.

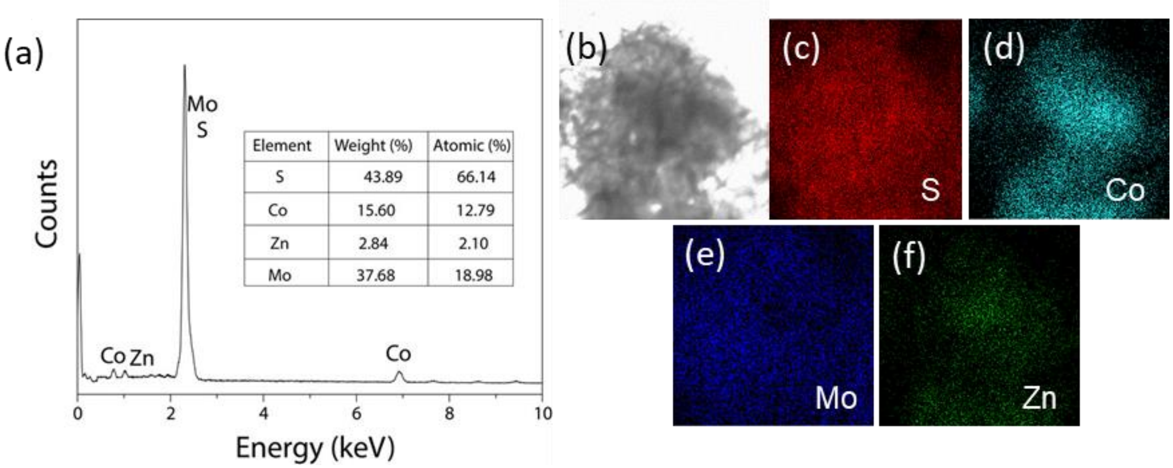

Figure S7. (a) TEM-EDX spectrum of MoS-CoS-Zn-1pot. Inset table shows weight and atomic percentages of Mo, Co, Zn and S elements in MoS-CoS-Zn-1pot structures. The contents of MoS-CoS-Zn-1pot are comparable to that of MoS-CoS-Zn. (b) STEM image and corresponding elemental mapping images showing the distribution of (c) S, (d) Co, (e) Mo and (f) Zn in MoS-CoS-Zn-1pot.

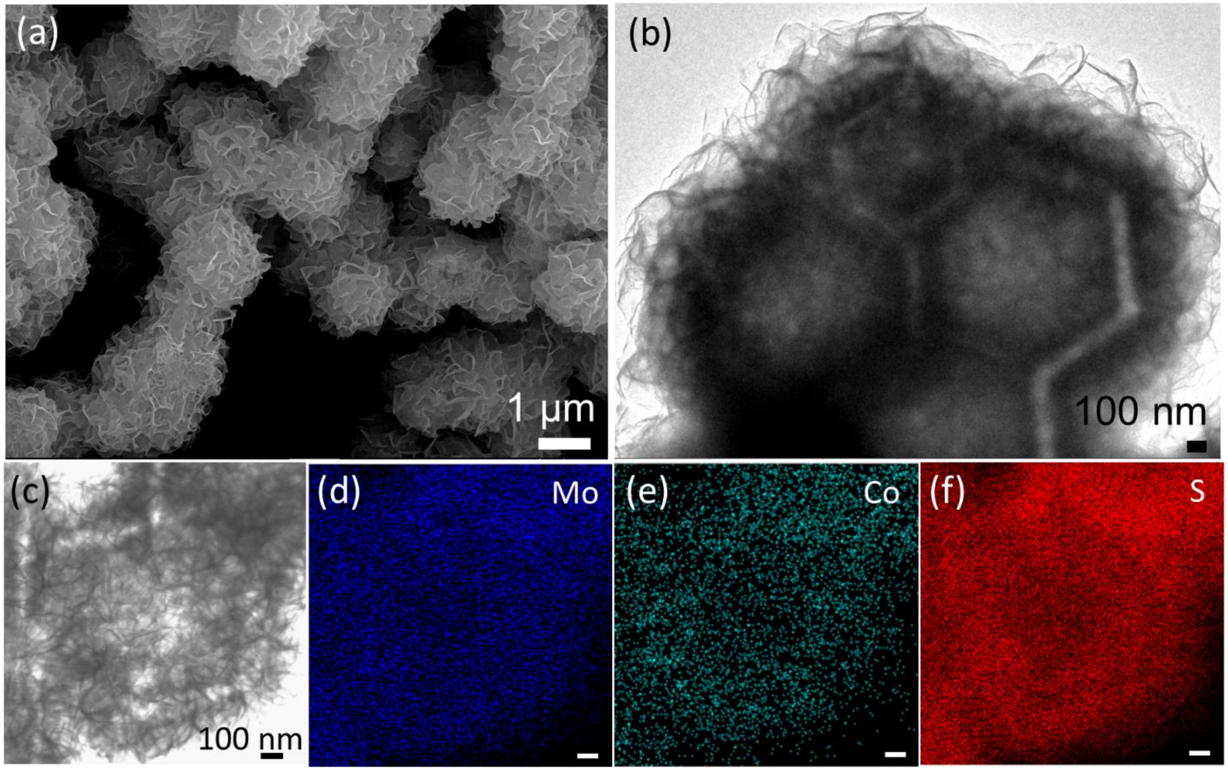

Figure S8. Structural characterization of MoS-CoS. (a) SEM and (b) TEM images of MoS-CoS. (c) High angle annular dark field (HAADF) scanning transmission electron microscopy (STEM) image of MoS-CoS, and corresponding EDX mapping images showing the homogeneous distribution of (d) Mo, (e) Co and (f) S elements in MoS-CoS. As seen in Figure S8a, MOF-derived MoS-CoS nanostructures are discrete nanoparticles similar to MoS-CoS-Zn, indicating the importance of exploitation of MOF templates for synthesis of uniformly distributed and non-aggregated particles. As confirmed by EDX mapping in Figure S8d-f, molybdenum, cobalt and sulfur elements are uniformly distributed over the entire structure.

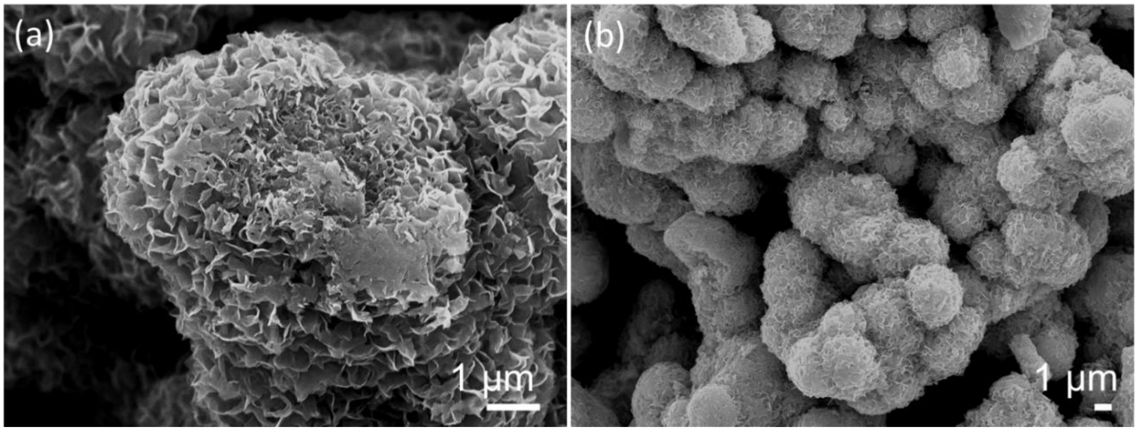

Figure S9. SEM images of pristine MoS_2 (MoS). (a) and (b) SEM images at different magnifications. As shown in Figure S9, MoS_2 possesses micro-sized spherical morphology surrounded by nanosheets. The morphology of MoS_2 structures are similar to that of MoS-CoS-Zn-1pot structures.

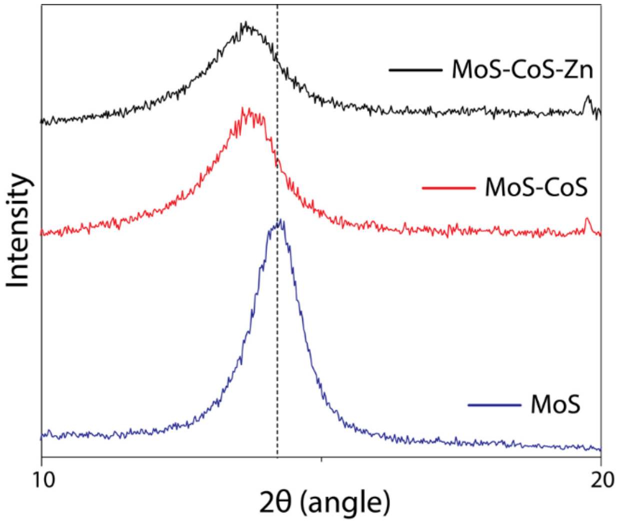

Figure S10. Zoomed-in X-ray diffraction patterns of MoS, MoS-CoS and MoS-CoS-Zn. Figure S10 shows the zoomed-in part of Figure 2a in the range of 2θ=10-20°, focusing the first peak of XRD. The dashed line indicates the first peak position of MoS, revealing the (002) plane. Noticeably, the (002) plane of MoS-CoS hybrid is slightly shifted (0.6°) to lower angle compared with pristine 2H-MoS_2 (MoS), indicating slight interlayer space expansion.

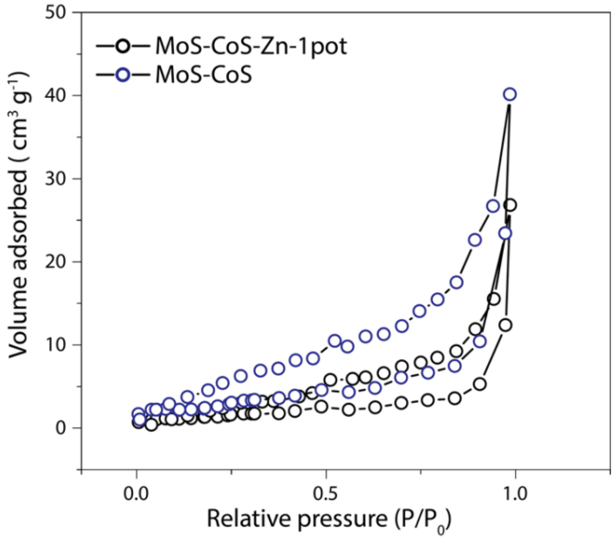

Figure S11. N_2 adsorption-desorption isotherms of MoS-CoS and MoS-CoS-Zn-1pot. Brunauer–Emmett–Teller (BET) specific surface area of MoS-CoS-Zn, is higher than that of MoS-CoS and MoS-CoS-Zn-1pot.

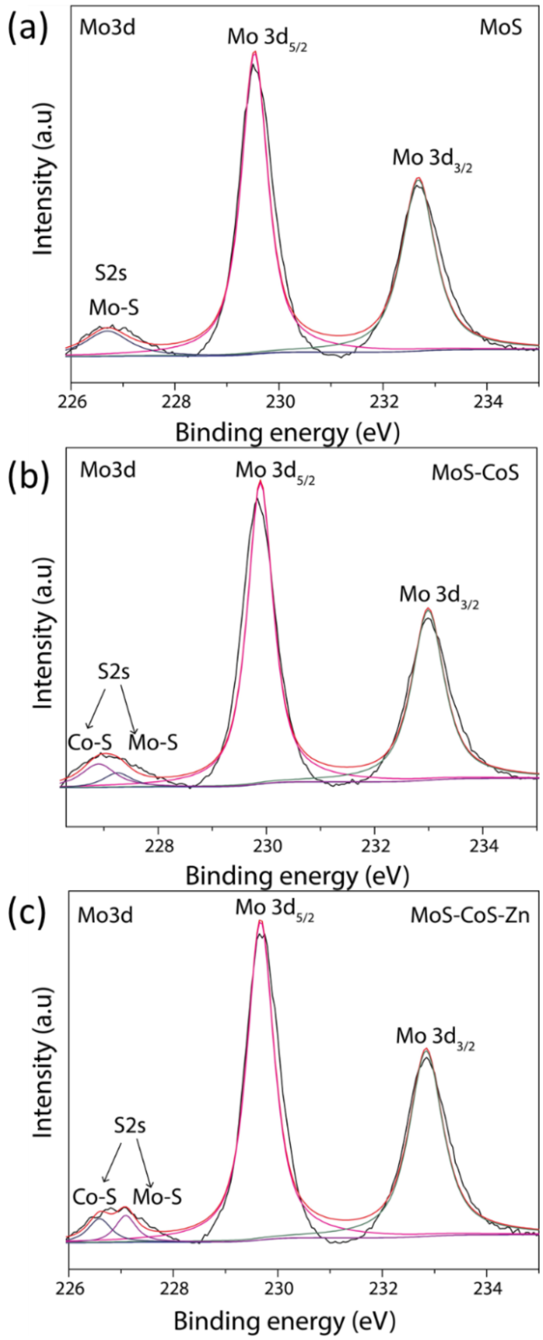

Figure S12. Deconvoluted core-level Mo3d spectra of the as-synthesized materials. Mo3d spectra of (a) MoS, (b) MoS-CoS and (c) MoS-CoS-Zn. The S2s peak is detected at nearby position to Mo3d. The S2s peak was deconvoluted into two peaks in XPS spectra of MoS-CoS and MoS-CoS-Zn, which correspond to the chemical sates of S element coordinated with cobalt and molybdenum ions.

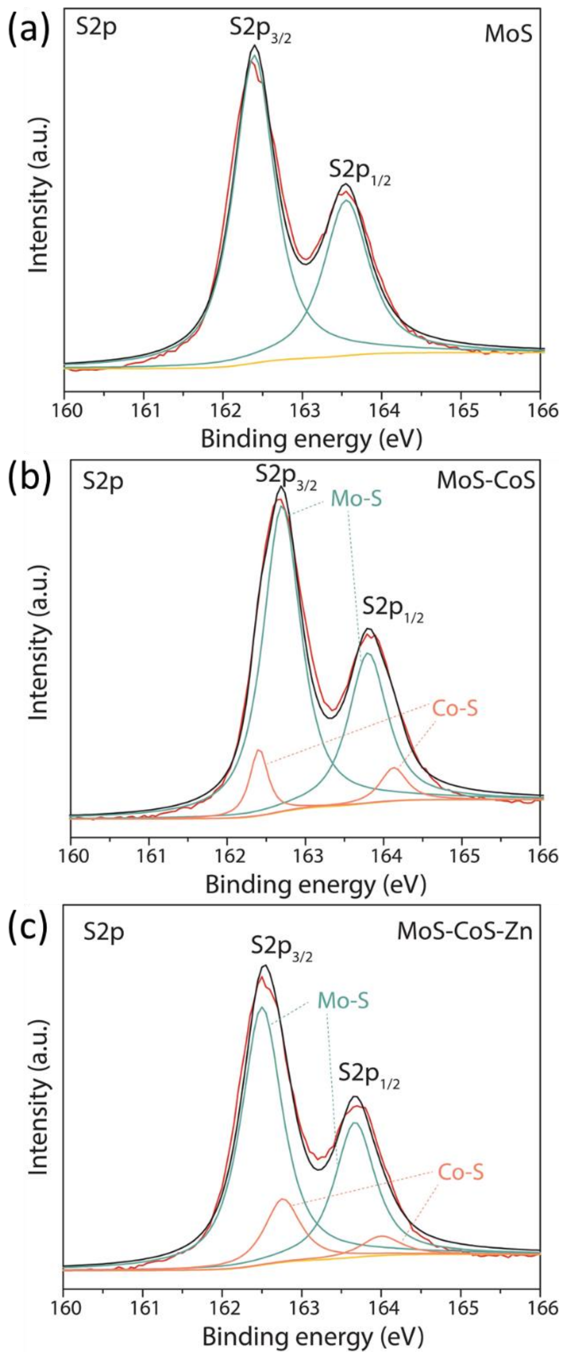

Figure S13. Deconvoluted core-level S2p spectra of the as-synthesized materials. S2p spectra of (a) MoS, (b) MoS-CoS and (c) MoS-CoS-Zn. The S2p peaks were deconvoluted into four peaks in XPS spectra of MoS-CoS and MoS-CoS-Zn, corresponding to $S2p_{1/2}$ and $S2p_{3/2}$ orbitals of divalent cobalt and molybdenum sulfide.

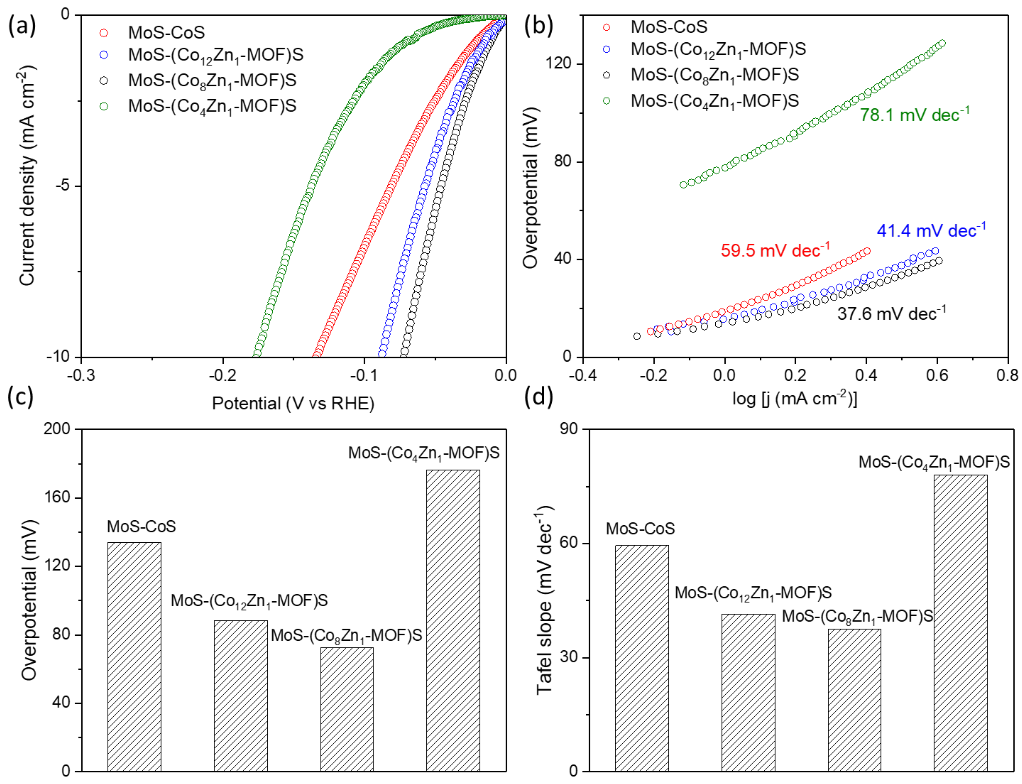

Figure S14. (a) HER polarization curves of MoS-CoS, MoS-($Co_{12}Zn_1$-MOF)S, MoS-(Co_8Zn_1-MOF)S and MoS-(Co_4Zn_1-MOF)S recorded at a scan rate of 5 mV s^{-1} in 0.5 M H_2SO_4. (b) Corresponding Tafel plots. Comparison of (c) overpotentials (at a current density of -10 mA cm^{-2}) and (d) Tafel slopes. To investigate the effect of Co/Zn ratio on the electrocatalytic performance of the MoS-CoS-Zn, MoS-($Co_{12}Zn_1$-MOF)S, MoS-(Co_8Zn_1-MOF)S and MoS-(Co_4Zn_1-MOF)S were prepared following the same synthesis procedure of MoS-CoS-Zn, except changing the Co/Zn molar ratios of MOF precursors. It should be noted that MoS-(Co_8Zn_1-MOF)S is the same as the MoS-CoS-Zn. Based on the SEM-EDX analysis, MoS-($Co_{12}Zn_1$-MOF)S and MoS-(Co_4Zn_1-MOF)S were found to exhibit Zn loadings of 0.8 and 3.7 atomic%, respectively. The atomic percentage of Zn in MoS-(Co_8Zn_1-MOF)S was already reported to be 2.75 (see Table S2), thereby allowing a rational Zn loading comparison. As shown in Figure S14a and S14c, MoS-(Co_8Zn_1-MOF)S shows the lowest overpotential of 72.6 mV at 10 mA cm^{-2}, exceeding that of the MoS-CoS (134 mV), MoS-($Co_{12}Zn_1$-MOF)S (88.5 mV) and MoS-(Co_4Zn_1-MOF)S (176.4 mV). Moreover, Tafel slope of MoS-(Co_8Zn_1-MOF)S is the smallest among the four electrodes (Figure S14b and S14d), which suggests that the optimum Co-Zn ratio is 8 to 1.

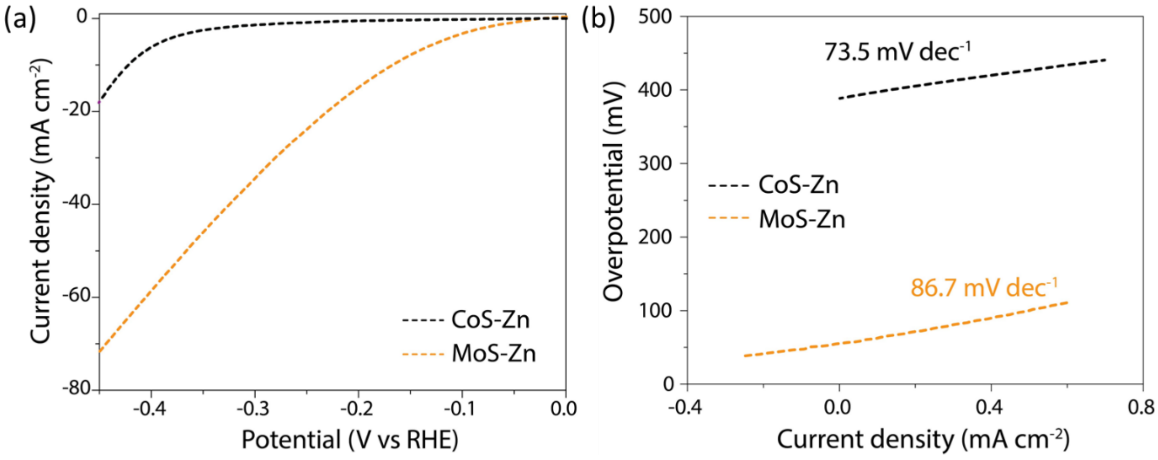

Figure S15. Electrocatalytic HER performance of MoS-Zn and CoS-Zn. (a) HER polarization curves of CoS-Zn and MoS-Zn recorded at a scan rate of 5 mVs^{-1} in 0.5 M aqueous H_2SO_4. (b) Corresponding Tafel plots of the electrocatalysts. As shown in Figure S15a, CoS-Zn possesses a high overpotential of 422 mV at a current density of 10 mA cm^{-2}, above which the current density increases rapidly. On the other hand, MoS-Zn requires an overpotential of 167 mV to drive the same cathodic current density. Figure S15b shows the Tafel slopes of CoS-Zn and MoS-Zn. CoS-Zn exhibits a slope of 73.5 mV dec^{-1}, which outperforms that of MoS-Zn (86.7 mV dec^{-1}), indicating that CoS-Zn provides faster increase of hydrogen generation rate with applied overpotential. However, neither MoS-Zn nor CoS-Zn satisfies the low overpotential-small Tafel slope requirement. Despite the smaller Tafel slope of CoS-Zn, it requires higher overpotential compared with MoS-Zn.

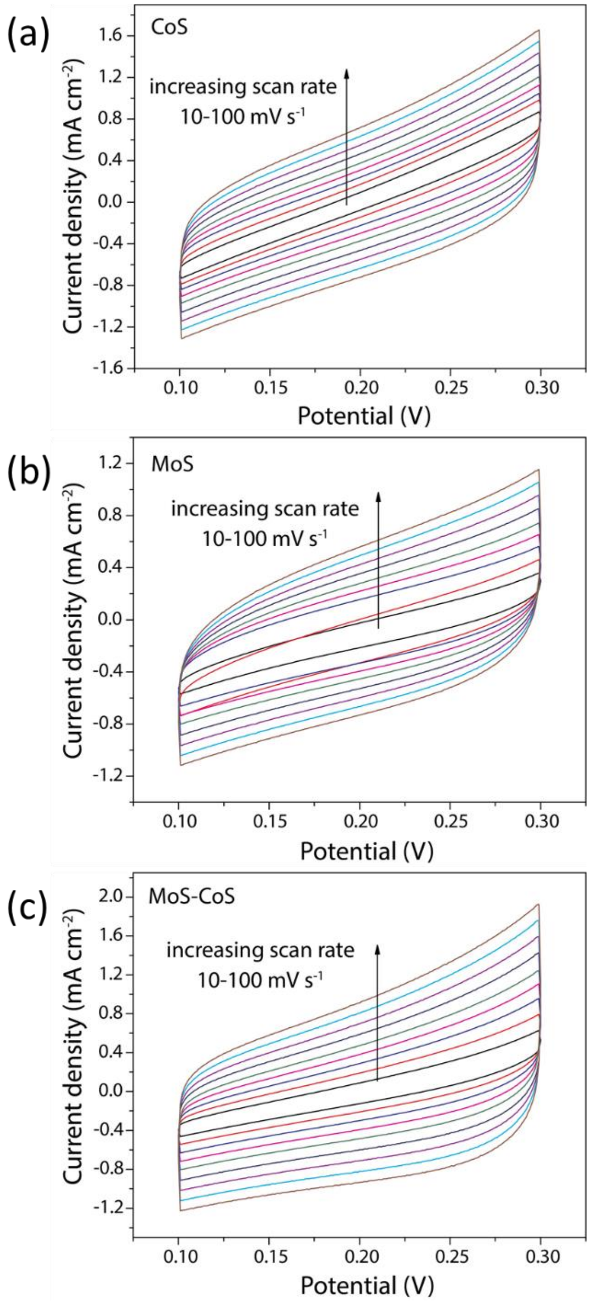

Figure S16. ECSA measurements of MoS, CoS, and MoS-CoS. Cyclic voltammograms measured in non-Faradaic region for (a) CoS, (b) MoS and (c) MoS-CoS at scan rates of 10, 20, 30, 40, 50, 60, 70, 80, 90, and 100 mVs^{-1} in the potential range within 0.1 - 0.3 V (vs RHE). The electrochemically active surface areas (ECSAs) of the electrocatalysts were assessed by cyclic voltammetry measurements in the double layer region, eliminating the effects of faradaic processes. Double layer capacitances of 13.4 mF cm^{-2}, 16.5 mF cm^{-2} and 19.8 mF cm^{-2} were obtained for CoS, MoS and MoS-CoS, respectively, by plotting the capacitive current densities at 0.2 V (vs RHE) as a function of scan rates.

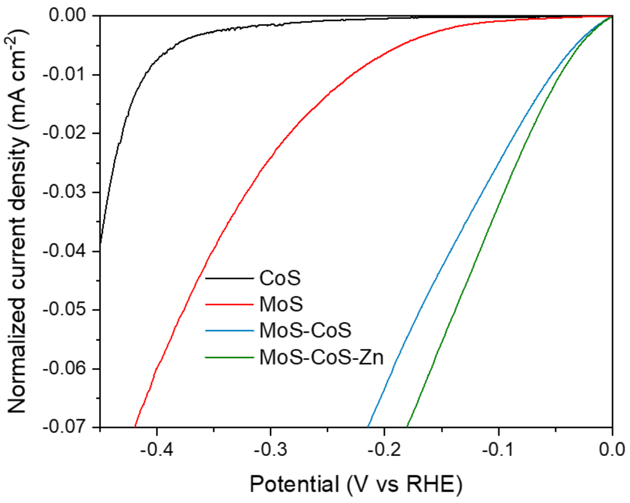

Figure S17. HER polarization curves of the electrocatalysts normalized by ECSA.

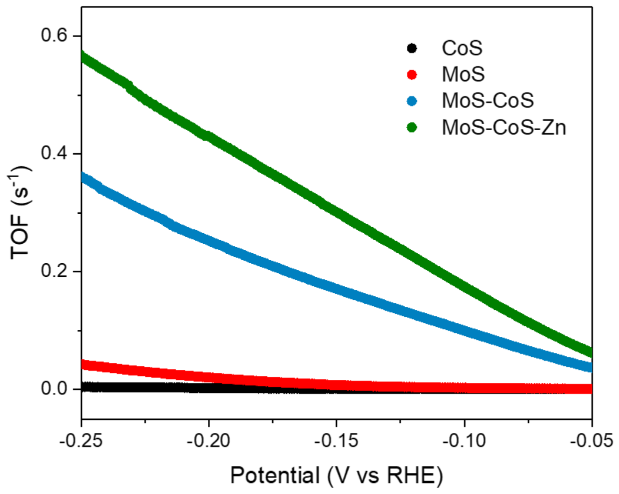

Figure S18. Calculated TOF values for the electrocatalysts.

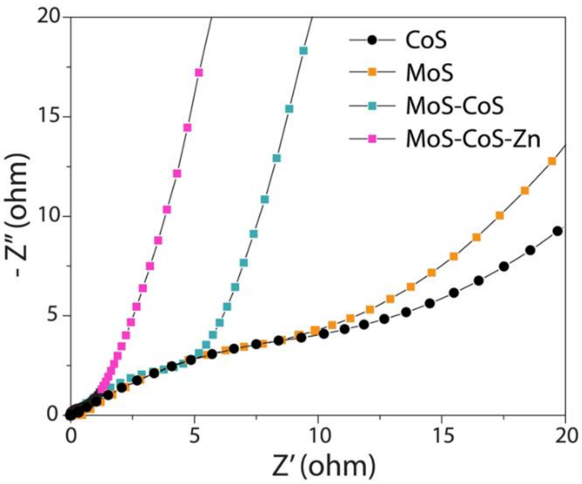

Figure S19. Electrochemical impedance spectra investigations of CoS, MoS, MoS-CoS and MoS-CoS-Zn. The electrochemical impedance spectra (EIS) were recorded at an overpotential of 100 mV in 0.5 M aqueous H_2SO_4. In all the Nyquist plots, a semicircle at high frequency region, and a linear plot at low frequency were observed, indicating the presence of charge transfer and mass transfer resistances, respectively. The Nyquist plots show that MoS-CoS-Zn has the smallest resistances, indicating superior HER kinetics.

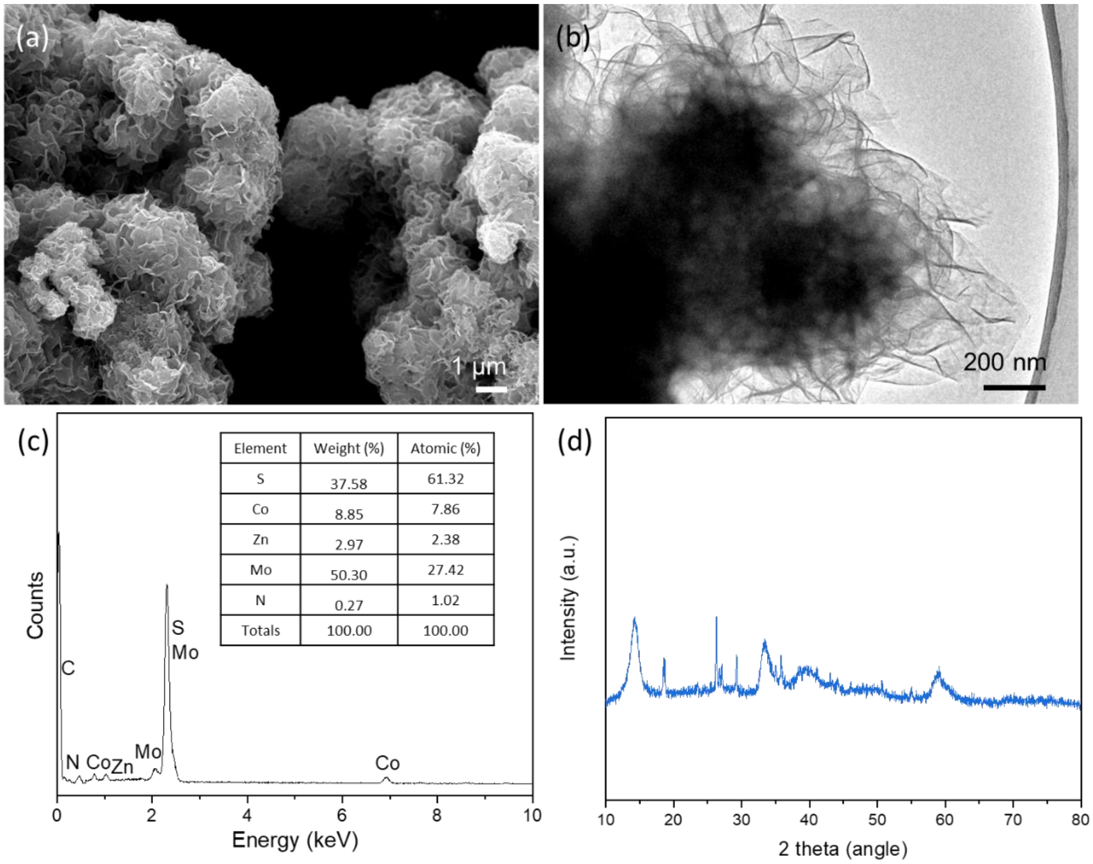

Figure S20. (a) SEM image, (b) TEM image, (c) SEM-EDX analysis and (d) XRD pattern of MoS-CoS-Zn electrocatalyst after 60 h continuous HER operation. The crystal structure and morphology of the MoS-CoS-Zn after 60 h continuous HER operation were investigated using SEM, TEM, XRD and EDX characterizations. As seen from (Figure S20a,b), the morphology of the MoS-CoS-Zn remains almost unchanged, retaining the original 3D microstructures that are composed of abundant 2D nanosheets. It should be noted that slight aggregation is due to the existence of Nafion, which was employed as binder during electrocatalyst preparation. Moreover, SEM-EDX analysis (Figure S20c) confirms the existence of Co, Mo, S, Zn and N in the post-HER MoS-CoS-Zn, and the composition is consistent with that of the pre-HER MoS-CoS-Zn (see Table S2), indicating compositional stability of the electrocatalyst. XRD pattern in Figure S20d also reveals existence of MoS_2 and CoS_2 (similar to the XRD pattern before stability test), suggesting that the crystal structure and phase of the electrocatalyst do not change during HER testing.

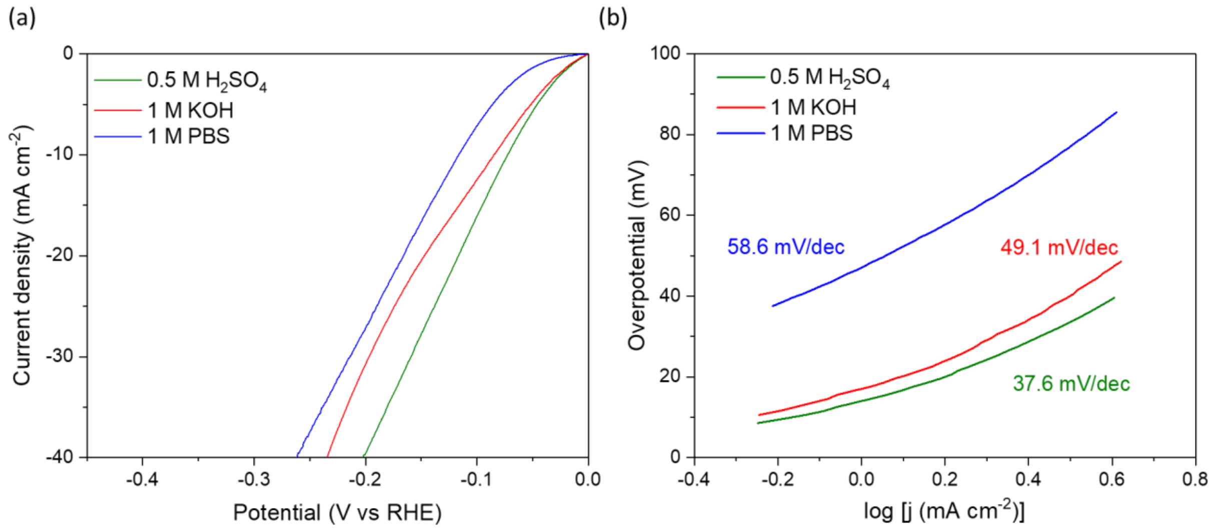

Figure S21. (a) HER polarization curves at a scan rate of 5 mV s^{-1} and (b) corresponding Tafel plots of MoS-CoS-Zn collected in 0.5 M H_2SO_4, 1 M KOH and 1 M PBS electrolytes.

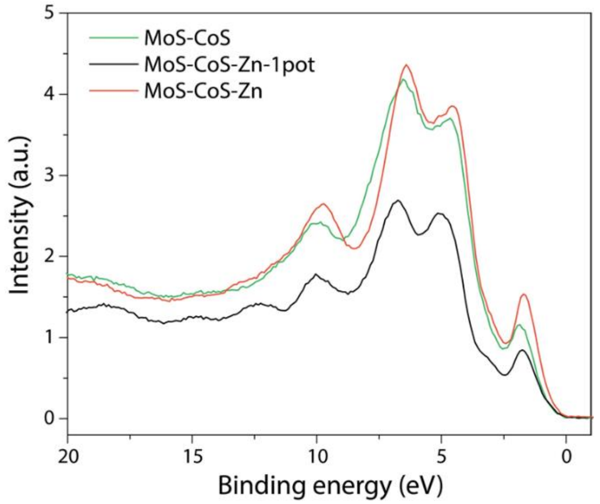

Figure S22. Overall UP spectra of MoS-CoS, MoS-CoS-Zn and MoS-CoS-Zn-1pot. MoS-CoS-Zn exhibits more metallic character compared with MoS-CoS and MoS-CoS-Zn-1pot as seen from the UPS spectra. Obviously, the peak close to the Fermi level becomes more intense with zinc-insertion (for MoS-CoS-Zn compared with MoS-CoS), which can be attributed to the more electrons around its Fermi level. Meanwhile, MoS-CoS-Zn-1pot has higher valence band maximum value (0.70 eV) than MoS-CoS-Zn.

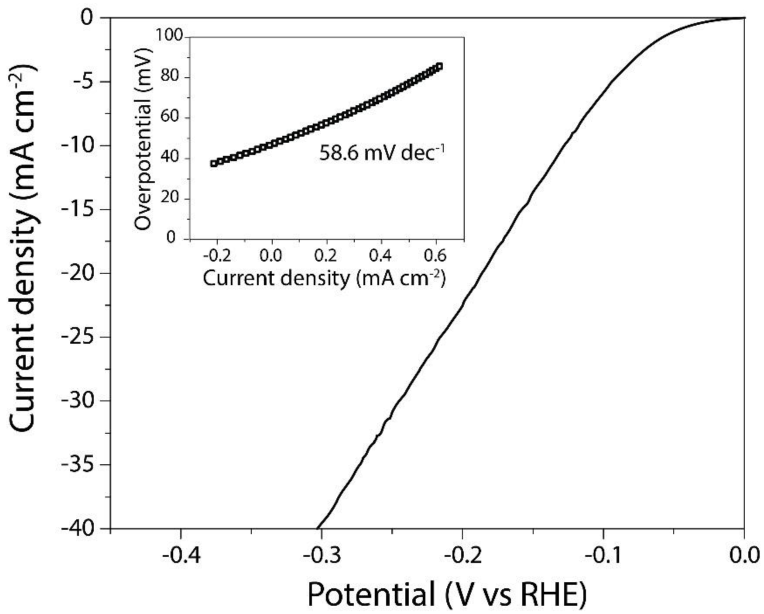

Figure S23. HER polarization curve and Tafel plot of MoS-CoS-Zn-1pot. Above figure presents the HER polarization curve of MoS-CoS-Zn-1pot obtained from linear sweep voltammetry measurements carried out at 5 mV s^{-1} in 0.5 M aqueous H_2SO_4. MoS-CoS-Zn-1pot requires an overpotential of 128 mV to drive a cathodic current density of 10 mA cm^{-2}. The inset is the corresponding Tafel plot, showing a Tafel slope of 58.6 mV dec^{-1}.

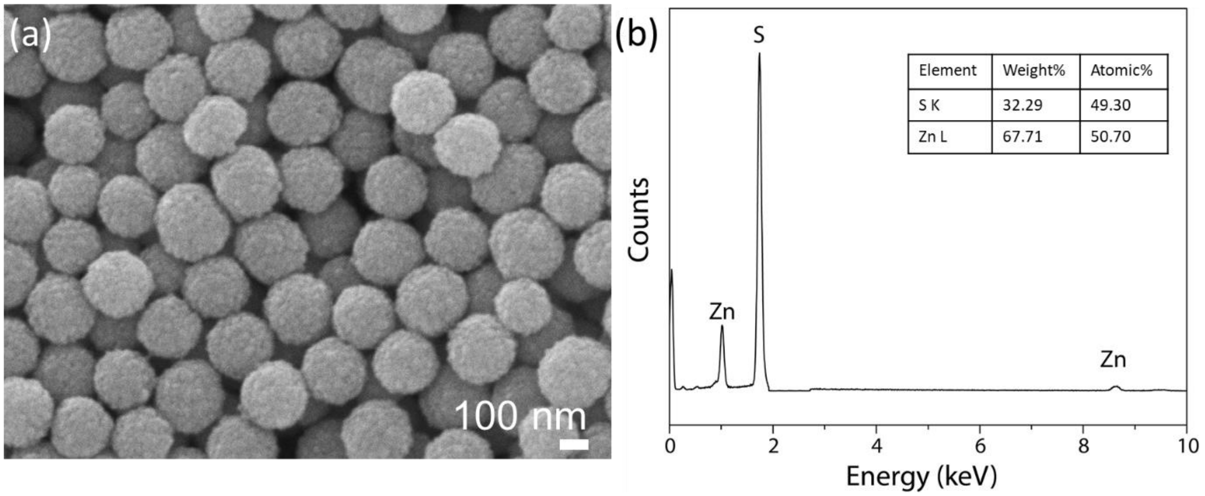

Figure S24. Characterization of ZnS. (a) SEM image and (b) EDX spectrum of ZnS nanospheres. The ZnS nanostructures were synthesized to be used in X-ray absorption near-edge structure spectroscopy analysis. As seen from Figure S24, Zn to S atomic ratio is ~1 to 1, indicating successful synthesis of zinc sulfide (ZnS).

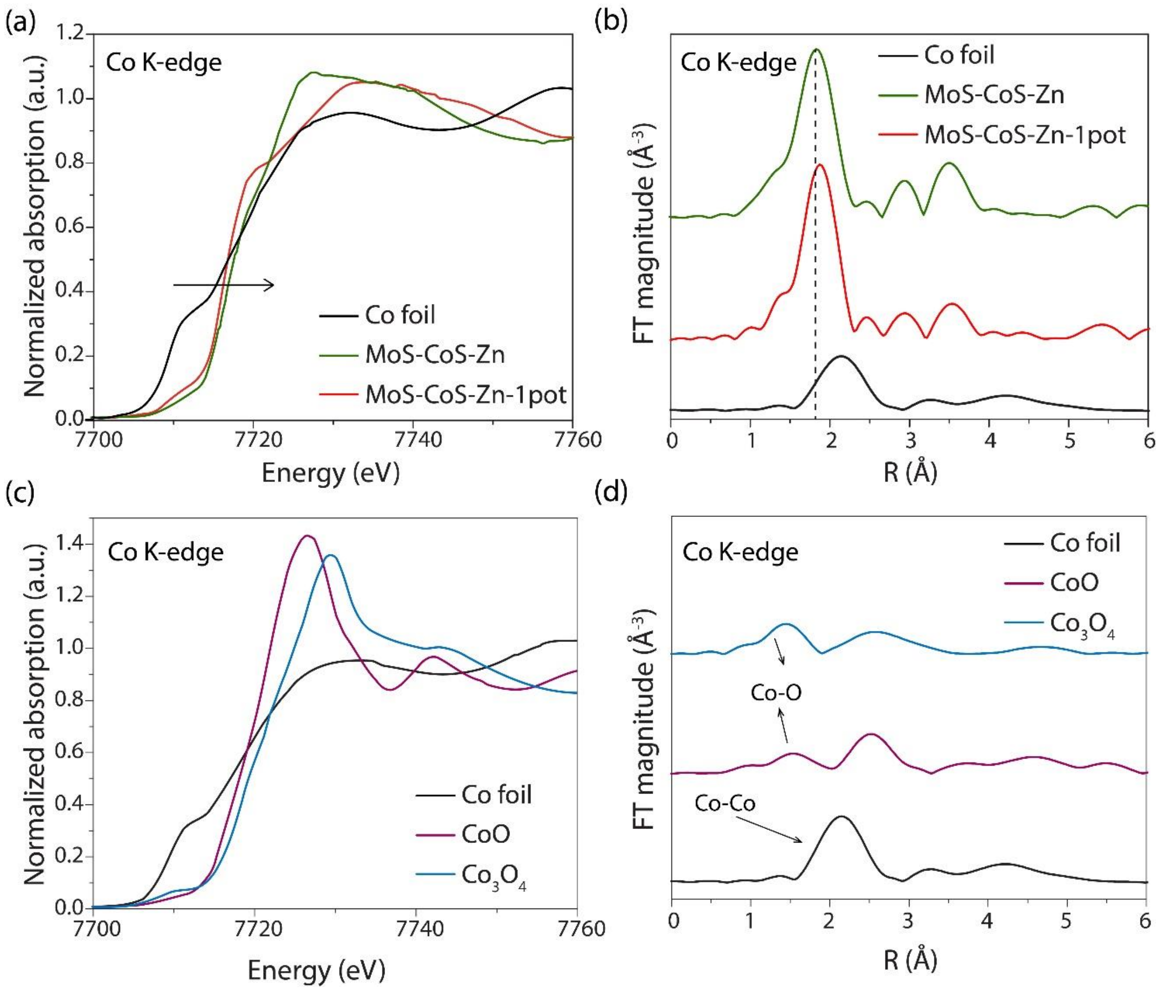

Figure S25. Structural and electronic properties of MoS-CoS-Zn in comparison to MoS-CoS-Zn-1pot based on Co K-edge XANES spectra: (a) Normalized Co K-edge XANES spectra of MoS-CoS-Zn, MoS-CoS-Zn-1pot and Co foil. (b) Co K-edge k^2-weighted EXAFS spectra of MoS-CoS-Zn, MoS-CoS-Zn-1pot and Co foil. Structural and electronic properties of cobalt oxides based on Co K-edge XANES spectra: (c) Normalized Co K-edge XANES spectra of CoO, Co_3O_4 and Co foil. (b) Co K-edge k^2-weighted EXAFS spectra of CoO, Co_3O_4 and Co foil. The Co K-edge XANES and EXAFS spectra were further investigated to reveal the local coordination environment of Co atoms in the framework of MoS_2 and Zn atoms. As shown in Figure S25a, MoS-CoS-Zn exhibits stronger white line intensity and more positive absorption edge compared with MoS-CoS-Zn-1pot. This result is consistent with the observations of Zn K-edge spectra, indicating that the valence state of cobalt in MoS-CoS-Zn is larger and possibly more electron transfer occurs towards molybdenum in MoS-CoS-Zn structure. The analysis of the first shell of MoS-CoS-Zn-1pot signifies a Co-S distance of 1.86 Å, which agrees well with the Co-S bond length in CoS_2 crystal structure. The Co-S coordination in MoS-CoS-Zn shifts to a lower R-position demonstrating that

the main coordination mode of Co varies slightly compared with MoS-CoS-Zn-1pot. Moreover, XANES and EXAFS studies for cobalt oxides (CoO and Co3O4) further confirm the existence of Co-S bonding in MoS-CoS-Zn and MoS-CoS-Zn-1pot. As seen from Figure S25c, Co K-edge XANES of cobalt oxides show an intense white line peak, which does not appear in the spectrum of MoS-CoS-Zn, indicating the distinct atomic and electronic difference between cobalt oxides and MoS-CoS-Zn. Besides, EXAFS curves of CoO and Co_3O_4 (Figure S25d) clearly show that they exhibit similar Co-O (~1.5 Å) distances, which is remarkably shorter relative to the MoS-CoS-Zn, confirming the sulfide phase.

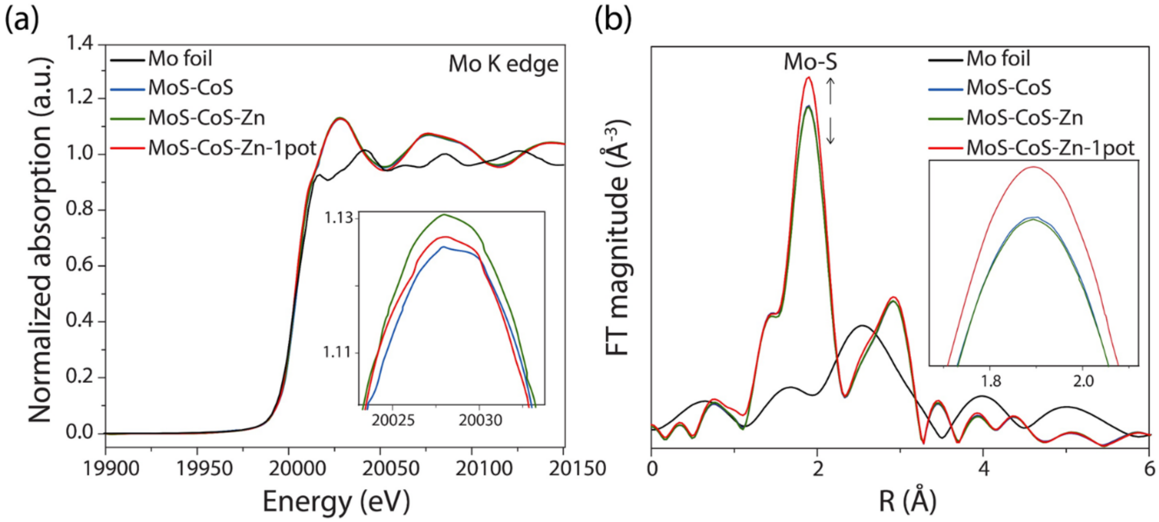

Figure S26. Structural and electronic properties of MoS-CoS-Zn in comparison to MoS-CoS-Zn-1pot and MoS-CoS based on Mo K-edge XANES spectra. (a) Normalized Mo K-edge XANES spectra of MoS-CoS-Zn, MoS-CoS-Zn-1pot, MoS-CoS and Mo foil. (b) Mo K-edge k^2-weighted EXAFS spectra of MoS-CoS-Zn, MoS-CoS-Zn-1pot, MoS-CoS and Mo foil. The insets show the zoomed-in view of the white line and Mo-S peak intensities.

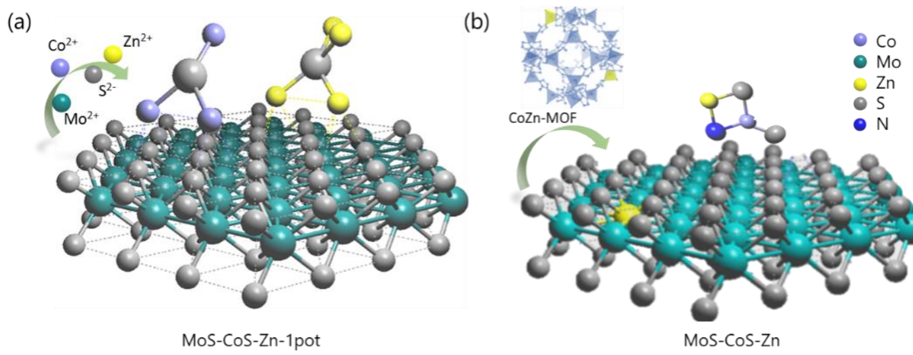

Figure S27. Predicted schematic models. Predicted schematic models of (a) MoS-CoS-Zn-1pot and (b) MoS-CoS-Zn.

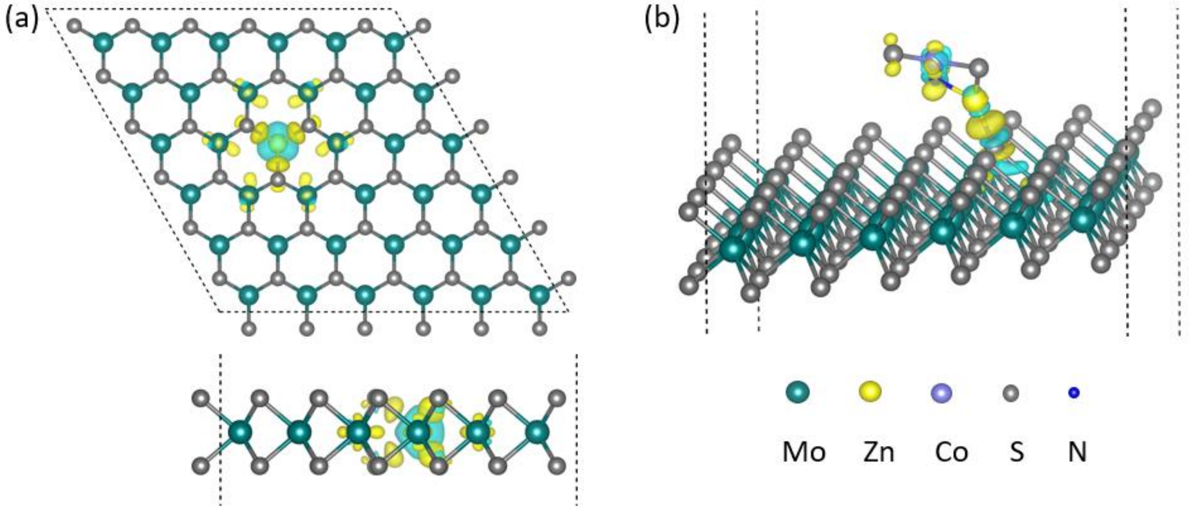

Figure S28. Differential charge density of Zn-MoS_2 (MoS-Zn) and CoS_2ZnN@Zn-MoS_2. (a) The top view (upper panel) and side view (bottom panel) of the charge density difference of the Zn doped MoS_2 (Zn-MoS_2 or MoS-Zn). (b) The charge density difference of the MoS_2 with a CoS_2ZnN cluster adsorbed (CoS_2ZnN@MoS_2). The charge density difference is defined as: $\Delta Q(X + MoS_2) = Q(X + MoS_2) - Q(MoS_2) - Q(X)$, where X is the Zn dopant in (a) or the adsorbed CoS_2ZnN cluster in (b). The isosurface with an isovalue of 0.002 eV/Å^3 is visualized in both (a) and (b), where the charge accumulation and depletion are denoted in yellow and cyan, respectively.

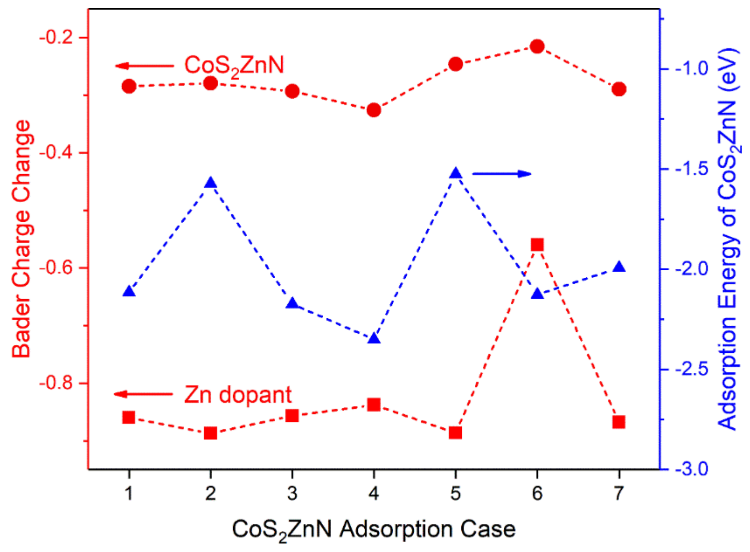

Figure S29. Bader charge and adsorption energy analysis. The Bader charge changes of CoS_2ZnN@Zn-MoS_2 (MoS-CoS-Zn) with seven possible CoS_2ZnN adsorption cases on Zn-MoS_2 (MoS-Zn) (see in Figure S30), and corresponding adsorption energies.

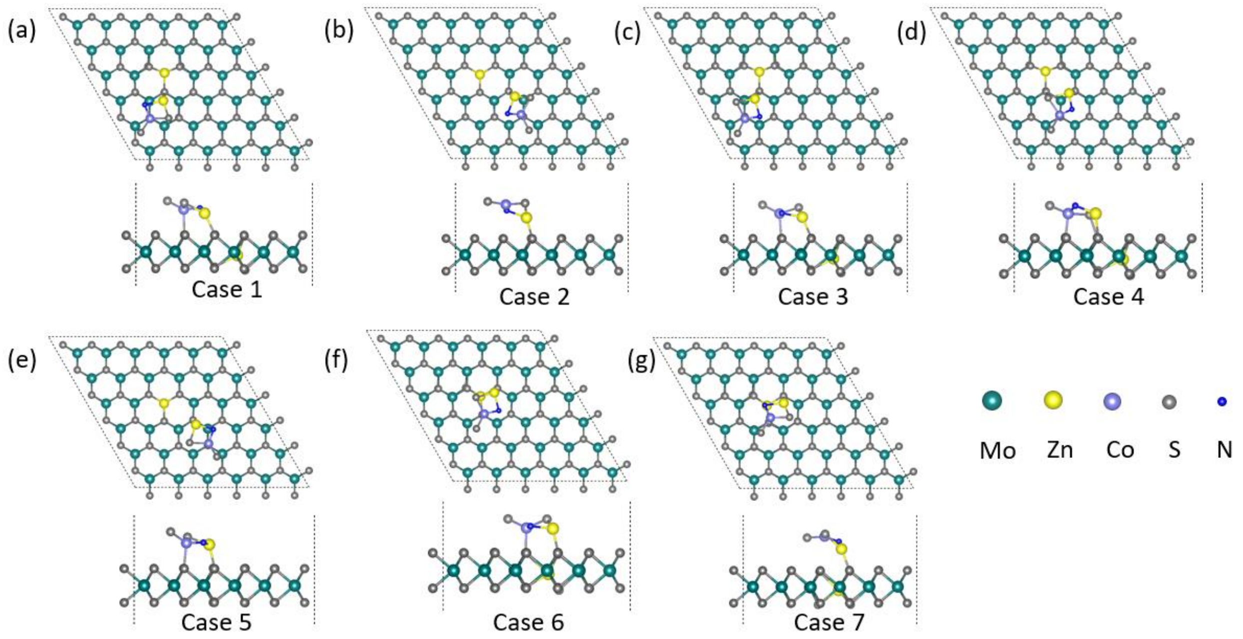

Figure S30. Optimized structures. Illustration of the optimized structures for seven possible CoS_2ZnN adsorption cases nearby the Zn dopant on the Zn-doped MoS_2 (Zn-MoS_2 or MoS-Zn) supercell.

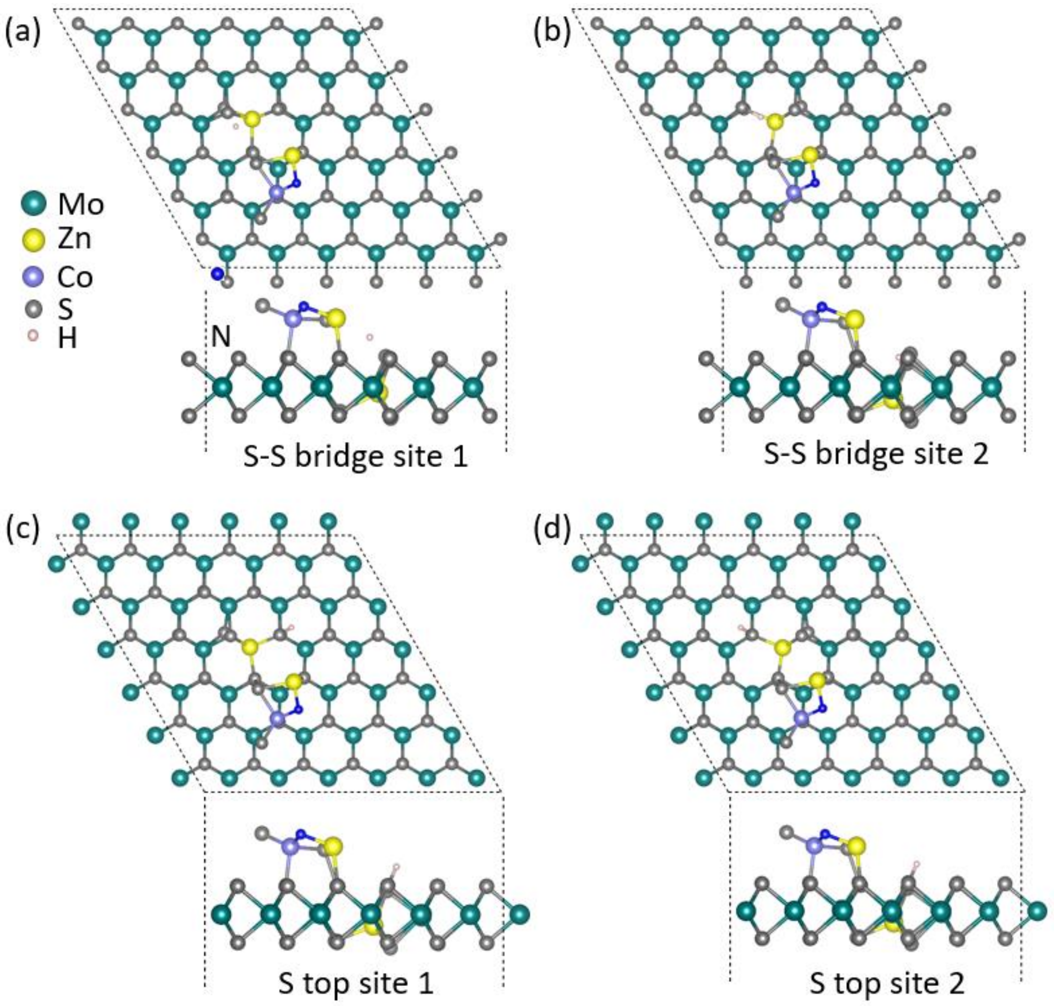

Figure S31. Optimized hydrogen adsorption sites. Hydrogen adsorption on (a) the S-S bridge site 1, (b) the S-S bridge site 2, (c) the S top site 1 and (d) the S top site 2 for the CoS_2ZnN adsorption case 4 (see in Figure S30). These hydrogen adsorption sites are also presented in Figure 5b.

Table S1. Molar percentages of Zn and Co in bimetallic organic frameworks obtained from SEM-EDX analysis. Bimetallic frameworks are named as Co_xZn_y-MOF. x and y represent molar ratios of zinc and cobalt precursors, respectively, in the bimetallic organic frameworks.

Initial Zn/Co ratio	MOF	Zn (molar %) in as-synthesized bimetallic MOF	Co (molar %) in as-synthesized bimetallic MOF	Final Zn/Co molar ratio
2/1	Co_1Zn_2-MOF	76	24	3.0
1/1	Co_1Zn_1-MOF	69	31	2.2
1/2	Co_2Zn_1-MOF	58	42	1.4
1/4	Co_4Zn_1-MOF	42	58	0.7
1/8	Co_8Zn_1-MOF	24	76	0.3
1/12	$Co_{12}Zn_1$-MOF	18	82	0.2

Table S2. Atomic and weight percentages of the constituting elements in MoS-CoS-Zn obtained from TEM-EDX.

Element	Weight%	Atomic%
**S**	40.71	63.98
**Co**	8.51	7.28
**Zn**	3.56	2.75
**Mo**	46.81	24.58
**N**	0.39	1.41
**Totals**	100.00	100.00

Table S3. HER activities of MoS_2-based electrocatalysts in acidic, alkaline and neutral media.

Reference	Catalyst	Overpotential (mV)	Tafel slope (mV/dec)
**Electrocatalytic activities in acidic electrolyte (Overpotentials at 10 mA cm^{-2})**			
**This work**	**CoS-MoS-Zn**	**72.6**	**37.6**
This work	MoS-CoS	134	59.5
This work	MoS	373	114.3
This work	MoS-Zn	167	86.7
This work	CoS	463	79.9

This work	CoS-Zn	422	73.5
Adv. Energy Mater. 2017, 7, 1602086 [16]	N-MoS_2	46	47
J. Am. Chem. Soc. 2017, 139, 15479 [17]	Zn-MoS_2	140	51
Energy Environ. Sci., 2015, 8, 1594 [18]	Pt-MoS_2	140	96
J. Am. Chem. Soc. 2015, 137, 7365 [19]	Au-MoS_2	260	71
ACS Appl. Mater. Interfaces 2017, 9, 33, 27715 [20]	N, F co-doped MoS_2	230	57
ACS Energy Lett. 2017, 2, 745 [21]	P-doped MoS_2	43	34
Nano Energy 53 (2018) 458 [22]	Ni single-atom decorated MoS_2	110	74
ACS Appl. Mater. Interfaces 2017, 9, 34, 28394 [23]	Co_9S_8/NC@MoS_2	117	68.8
Adv. Funct. Mater. 2017, 27, 1602699 [24]	CoS_2@MoS_2/RGO	98	37.4
Chem. Mater. 2017, 29, 5566 [25]	Co_3S_4@MoS_2	210	88
ACS Sustainable Chem. Eng. 2018, 6, 12961 [26]	CoSx@MoS2 Microcubes	239	103
Angew. Chem. Int. Ed. 2015, 54, 7395 [27]	C@MoS2 nanoboxes	165	55
Adv. Mater. 2016, 28, 9006 [28]	Ni–Co–MoS_2 nanoboxes MoS_2	155	51
Adv. Funct. Mater. 2018, 28, 1707564 [29]	MoS_2/ N-doped graphdiyne	186	63
ACS Catal. 2018, 8, 8107 [30]	Dual-Modified MoS_2 with Nanoporous Ni and Graphene	205	71.3
ChemElectroChem 2018, 5, 3953 [31]	CoS/NC@MoS_2 hollow spheres	77	67
**Electrocatalytic activities in alkaline electrolyte (Overpotentials at 10 mA cm^{-2})**			

**This work**	**CoS-MoS-Zn**	**85.4**	**49.1**
Energy Environ. Sci., 2016, 9, 2789 [32]	Ni-MoS_2	98	60
	Co-MoS_2	203	158
	Fe-MoS_2	163	181
ACS Catal. 2018, 8, 7585 [33]	N, Mn co-doped MoS_2	66	50
Nano Energy 53 (2018) 458 [22]	Ni single-atom decorated MoS_2	95	75
Adv. Sci. 2018, 5, 1700644 [34]	MoS_2-Nickel Hydr(oxy)oxide	73	77
ACS Appl. Mater. Interfaces 2017, 9, 34, 28394 [23]	Co_9S_8/NC@MoS_2	67	60.3
ACS Nano 2018, 12, 4565 [35]	MoS_2 Confined $Co(OH)_2$	89	53
ACS Catal. 2018, 8, 4612 [36]	O-CoMoS	97	70
Adv. Mater. 2018, 30, 1801450 [37]	Co-MoS2/BCCF-21	48	52
ACS Catal. 2017, 7, 2357–2366 [38]	MoS_2-Ni_3S_2	98	61
ACS Appl. Mater. Interfaces 2018, 10, 2, 1752 [39]	MoS_2/Ni_3S_2/Ni	76	56
Adv. Energy Mater. 2019, 9, 1802553 [40]	S-MoS_2@C	155	99
**Electrocatalytic activities in neutral electrolyte (Overpotentials at 10 mA cm^{-2})**			
**This work**	**CoS-MoS-Zn**	**116.2**	**58.6**
ACS Catal. 2018, 8, 7585 [33]	N, Mn co-doped MoS_2	70	65
ACS Appl. Mater. Interfaces 2017, 9, 34, 28394 [23]	Co_9S_8/NC@MoS_2	261	126.1
Adv. Sci. 2018, 5, 1700644 [34]	MoS_2-Nickel Hydr(oxy)oxide	153	106

References

[1] X. Yu, O. Wilhelmi, H. O. Moser, S. V. Vidyaraj, X. Gao, A. T. Wee, T. Nyunt, H. Qian, H. Zheng, *J. Electron. Spectrosc. Relat. Phenom.* **2005**, *144*, 1031.

[2] Y. Du, Y. Zhu, S. Xi, P. Yang, H. O. Moser, M. B. Breese, A. Borgna, *J. Synchrotron Radiat.* **2015**, *22*, 839.

[3] J. P. Perdew, K. Burke, M. Ernzerhof, *Phys. Rev. Lett.* **1996**, *77*, 3865.

[4] G. Kresse, J. Hafner, *Phys. Rev. B: Condens. Matter Mater. Phys.* **1993**, *48*, 13115.

[5] G. Kresse, J. Hafner, *Phys. Rev. B: Condens. Matter Mater. Phys.* **1993**, *47*, 558.

[6] G. Kresse, D. Joubert, *Phys. Rev. B: Condens. Matter Mater. Phys.* **1999**, *59*, 1758.

[7] S. Grimme, J. Antony, S. Ehrlich, H. Krieg, *J. Chem. Phys.* **2010**, *132*, 154104.

[8] S. Grimme, S. Ehrlich, L. Goerigk, *J. Comput. Chem.* **2011**, *32*, 1456.

[9] G. Makov, M. Payne, *Phys. Rev. B: Condens. Matter Mater. Phys.* **1995**, *51*, 4014.

[10] J. Neugebauer, M. Scheffler, *Phys. Rev. B: Condens. Matter Mater. Phys.* **1992**, *46*, 16067.

[11] A. Ramasubramaniam, *Phys. Rev. B: Condens. Matter Mater. Phys.* **2012**, *86*, 115409.

[12] B. Hinnemann, P. G. Moses, J. Bonde, K. P. Jørgensen, J. H. Nielsen, S. Horch, I. Chorkendorff, J. K. Nørskov, *J. Am. Chem. Soc.* **2005**, *127*, 5308.

[13] J. K. Nørskov, T. Bligaard, A. Logadottir, J. R. Kitchin, J. G. Chen, S. Pandelov, U. Stimming, *J. Electrochem. Soc.* **2005**, *152*, J23.

[14] P. Atkins, J. De Paula, *Elements of physical chemistry*, Oxford University Press, USA, 2013.

[15] A. F. Gross, E. Sherman, J. J. Vajo, *Dalton Trans.* **2012**, *41*, 5458.

[16] W. Xiao, P. Liu, J. Zhang, W. Song, Y. P. Feng, D. Gao, J. Ding, *Adv. Energy Mater.* **2017**, *7*, 1602086.

[17] Y. Shi, Y. Zhou, D.-R. Yang, W.-X. Xu, C. Wang, F.-B. Wang, J.-J. Xu, X.-H. Xia, H.-Y. Chen, *J. Am. Chem. Soc.* **2017**, *139*, 15479.

[18] J. Deng, H. Li, J. Xiao, Y. Tu, D. Deng, H. Yang, H. Tian, J. Li, P. Ren, X. Bao, *Energy Environ. Sci.* **2015**, *8*, 1594.

[19] Y. Shi, J. Wang, C. Wang, T.-T. Zhai, W.-J. Bao, J.-J. Xu, X.-H. Xia, H.-Y. Chen, *J. Am. Chem. Soc.* **2015**, *137*, 7365.

[20] Y. Wang, S. Liu, X. Hao, J. Zhou, D. Song, D. Wang, L. Hou, F. Gao, *ACS Appl. Mater. Interfaces* **2017**, *9*, 27715.

[21] P. Liu, J. Zhu, J. Zhang, P. Xi, K. Tao, D. Gao, D. Xue, *ACS Energy Lett.* **2017**, *2*, 745.

[22] Q. Wang, Z. L. Zhao, S. Dong, D. He, M. J. Lawrence, S. Han, C. Cai, S. Xiang, P. Rodriguez, B. Xiang, *Nano Energy* **2018**, *53*, 458.

[23] H. Li, X. Qian, C. Xu, S. Huang, C. Zhu, X. Jiang, L. Shao, L. Hou, *ACS Appl. Mater. Interfaces* **2017**, *9*, 28394.

[24] Y. Guo, L. Gan, C. Shang, E. Wang, J. Wang, *Adv. Funct. Mater.* **2017**, *27*, 1602699.

[25] Y. Guo, J. Tang, H. Qian, Z. Wang, Y. Yamauchi, *Chem. Mater.* **2017**, *29*, 5566.

[26] L. Yang, L. Zhang, G. Xu, X. Ma, W. Wang, H. Song, D. Jia, *ACS Sustain. Chem. Eng.* **2018**, *6*, 12961.

[27] X.-Y. Yu, H. Hu, Y. Wang, H. Chen, X. W. Lou, *Angew. Chem. Int. Ed.* **2015**, *54*, 7395.

[28] X. Y. Yu, Y. Feng, Y. Jeon, B. Guan, X. W. Lou, U. Paik, *Adv. Mater.* **2016**, *28*, 9006.

[29] H. Yu, Y. Xue, L. Hui, C. Zhang, Y. Zhao, Z. Li, Y. Li, *Adv. Funct. Mater.* **2018**, *28*, 1707564.

[30] L. X. Chen, Z. W. Chen, Y. Wang, C. C. Yang, Q. Jiang, *ACS Catal.* **2018**, *8*, 8107.

[31] Y. Zhao, M. Bi, F. Qian, P. Zeng, M. Chen, R. Wang, Y. Liu, Y. Ding, Z. Fang, *ChemElectroChem* **2018**, *5*, 3953.

[32] J. Zhang, T. Wang, P. Liu, S. Liu, R. Dong, X. Zhuang, M. Chen, X. Feng, *Energy Environ. Sci.* **2016**, *9*, 2789.

[33] T. Sun, J. Wang, X. Chi, Y. Lin, Z. Chen, X. Ling, C. Qiu, Y. Xu, L. Song, W. Chen, *ACS Catal.* **2018**, *8*, 7585.

[34] X. Zhang, Y. Liang, *Adv. Sci.* **2018**, *5*, 1700644.
[35] Y. Luo, X. Li, X. Cai, X. Zou, F. Kang, H.-M. Cheng, B. Liu, *ACS Nano* **2018**, *12*, 4565.
[36] J. Hou, B. Zhang, Z. Li, S. Cao, Y. Sun, Y. Wu, Z. Gao, L. Sun, *ACS Catal.* **2018**, *8*, 4612.
[37] Q. Xiong, Y. Wang, P.-F. Liu, L.-R. Zheng, G. Wang, H.-G. Yang, P.-K. Wong, H. Zhang, H. Zhao, *Adv. Mater.* **2018**, *30*, 1801450.
[38] Y. Yang, K. Zhang, H. Lin, X. Li, H. C. Chan, L. Yang, Q. Gao, *ACS Catal.* **2017**, *7*, 2357.
[39] J. Cao, J. Zhou, Y. Zhang, Y. Wang, X. Liu, *ACS Appl. Mater. Interfaces* **2018**, *10*, 1752.
[40] Q. Xu, Y. Liu, H. Jiang, Y. Hu, H. Liu, C. Li, *Adv. Energy Mater.* **2019**, *9*, 1802553.
